# Validation of Reference Genes for Normalization of Relative qRT-PCR Studies in Papillary Thyroid Carcinoma

**DOI:** 10.1038/s41598-019-49247-1

**Published:** 2019-10-23

**Authors:** S. Adeleh Razavi, Mandana Afsharpad, Mohammad Hossein Modarressi, Maryam Zarkesh, Parichehreh Yaghmaei, Shirzad Nasiri, S. Mohammad Tavangar, Hanieh Gholami, Afsoon Daneshafrooz, Mehdi Hedayati

**Affiliations:** 1 Cellular and Molecular Endocrine Research Center, Research Institute for Endocrine Sciences, Shahid Beheshti University of Medical Sciences, Tehran, Iran; 2 Department of Research and Development (R&D), Saeed Pathobiology & Genetics Laboratory, Tehran, Iran; 3 Department of Biology, Faculty of Basic Sciences, Science and Research Branch, Islamic Azad University, Tehran, Iran; 4 Cancer Control Research Center, Cancer Control Foundation, Iran University of Medical Sciences, Tehran, Iran; 5 Department of Medical Genetics, School of Medicine, Tehran University of Medical Sciences, Tehran, Iran; 6 Department of Surgery, Shariati Hospital, School of Medicine, Tehran University of Medical Sciences, Tehran, Iran; 7 Department of Pathology, Shariati Hospital, School of Medicine, Tehran University of Medical Sciences, Tehran, Iran; 8 Endocrine Physiology Research Center, Research Institute for Endocrine Sciences, Shahid Beheshti University of Medical Sciences, Tehran, Iran

**Keywords:** Thyroid cancer, Gene expression

## Abstract

Quantitative reverse transcription polymerase chain reaction (qRT-PCR) in thyroid tumors require accurate data normalization, however, there are no sufficient studies addressing the suitable reference genes for gene expression analysis in malignant and normal thyroid tissue specimens. The purpose of this study was to identify valid internal control genes for normalization of relative qRT-PCR studies in human papillary thyroid carcinoma tissue samples. The expression characteristics of 12 candidate reference genes (GAPDH, ACTB, HPRT1, TBP, B2M, PPIA, 18SrRNA, HMBS, GUSB, PGK1, RPLP0, and PGM1) were assessed by qRT-PCR in 45 thyroid tissue samples (15 papillary thyroid carcinoma, 15 paired normal tissues and 15 multinodular goiters). These twelve candidate reference genes were selected by a systematic literature search. GeNorm, NormFinder, and BestKeeper statistical algorithms were applied to determine the most stable reference genes. The three algorithms were in agreement in identifying GUSB and HPRT1 as the most stably expressed genes in all thyroid tumors investigated. According to the NormFinder software, the pair of genes including ‘GUSB and HPRT1’ or ‘GUSB and HMBS’ or ‘GUSB and PGM1’ were the best combinations for selection of pair reference genes. The optimal number of genes required for reliable normalization of qPCR data in thyroid tissues would be three according to calculations made by GeNorm algorithm. These results suggest that GUSB and HPRT1 are promising reference genes for normalization of relative qRT-PCR studies in papillary thyroid carcinoma.

## Introduction

Among the methods used for gene expression studies including Northern blotting, microarrays, serial analysis of gene expression (SAGE), ribonuclease protection assays (RPAs) and quantitative reverse transcription polymerase chain reaction (qRT-PCR), the last one has been employed more than the others because of its sensitivity, specificity and low template requirements^1^. qRT-PCR gives more quantitative results and it is also easy and more convenient to use when compared to other methods. In the relative approach of qRT-PCR, it is necessary to apply a normalizing gene as an internal control to correct the differences between the compared samples in order to achieve the most accurate and reliable results^2^. Normalizing genes usually are the basic metabolism genes named housekeeping genes (HKGs) that are frequently used in studies. However, a single reference gene cannot be used for all experiments in all conditions because contrary to expectations, HKGs expression levels are affected by the specimen, method, experiment, and environment conditions. Thus, every specific experiment will require selecting the best reference gene to obtain accurate and reliable results^3^.

Currently, many cancer researchers investigate tumor marker genes to identify the pathological patterns of diseases and find much needed information about the predicting of the tumor’s behavior. However, expression patterns of HKGs may vary in different malignant tissues, different malignant cell subtypes, or even in the same type of carcinoma. This illustrates the difference in the personalized metabolism drivers of cancer cells when different cases are compared^2^.

Although the incidence rate of thyroid cancer is currently low but increasing, and it is estimated that it will be doubled and become the third most common cancer in women by 2019^4^. There are four main histopathological types of thyroid cancers including papillary thyroid carcinoma (PTC), follicular thyroid carcinoma (FTC), anaplastic thyroid carcinoma (ATC) with the origin of thyroid follicular cells and medullary thyroid carcinoma (MTC) with the origin of thyroid parafollicular cells. PTC is the most common and least aggressive histological type of thyroid cancers that its annual incidence rate is 0.29 per 100,000 persons in Iran^5^.

The most important diagnostic method for thyroid cancers is fine-needle aspiration biopsy (FNAB), however, 15–30% of thyroid FNABs are cytologically indeterminate^6^. For overcoming this problem, molecular based techniques are suggested to discover new molecular markers discriminating malignancy from benignity^7^. One of the most common methods for the investigation of thyroid tumor markers is qRT-PCR; however, there are no sufficient studies introducing the suitable reference genes for gene expression analysis in malignant and normal thyroid tissue samples. Definitely, without validation of reference genes, the results of gene expression studies in thyroid cancers are less reliable due to such unexpected behavior of HKGs. Therefore it is essential to determinate the genes with the highest level of expression stability for the improvement of relative qRT-PCR in further thyroid cancer research. The purpose of this study was to identify valid internal control genes for normalization of relative qRT-PCR studies in human PTC tissue samples.

## Results

### Quality of RNA samples

A260 and A280 were used to assess the concentration and purity of the isolated RNA. The mean and standard deviation (mean (ng/µl) ± SD) of A260/A280 ratio in PTC, paired normal tissues (PNT), and multinodular goiters (MNG) groups were 1.85 ± 0.15, 1.80 ± 0.12 and 1.93 ± 0.09 respectively.

### Amplification efficiency and specificity

To determine the amplification efficiency, serial dilutions of several cDNA templates were run for all 12 paired primers, and the results were used to depict a standard curve. The equation of the linear regression line, along with Pearson’s correlation coefficient (r) and the coefficient of determination (R^2^), confirmed the qPCR optimization (Table 1, Supplemental Fig. 1). The melt-curve analysis of all 12 qPCR assays exhibited a single signal peak represented the specific product (Supplemental Fig. 2).

**Table 1:** Standard curve results of selected candidate reference genes: M; slope, B; intercept, r; Pearson’s correlation coefficient, R^2^; coefficient of determination, E; amplification efficiency, %E; amplification efficiency percentage.

Gene	M	B	r	R^2^	E	%E
GAPDH	−3.15	32.48	0.991	0.982	2.08	%108
ACTB	−3.34	26.07	0.997	0.994	1.99	%99
HPRT1	−3.44	34.39	0.992	0.984	1.95	%95
TBP	−3.04	32.74	0.999	0.998	2.13	%113
B2M	−3.57	28.81	0.999	0.998	1.91	%91
PPIA	−3.29	37.03	0.998	0.996	2.01	%101
18SrRNA	−3.33	21.61	0.994	0.988	2.00	%100
HMBS	−3.53	36.20	0.998	0.997	1.92	%92
GUSB	−3.13	26.45	0.994	0.988	2.08	%108
PGK1	−3.38	36.40	0.999	0.997	1.98	%98
RPLP0	−3.34	33.93	0.977	0.955	1.99	%99
PGM1	−3.59	35.03	0.963	0.927	1.90	%90

### Candidate reference genes expression

**Figure 1:**
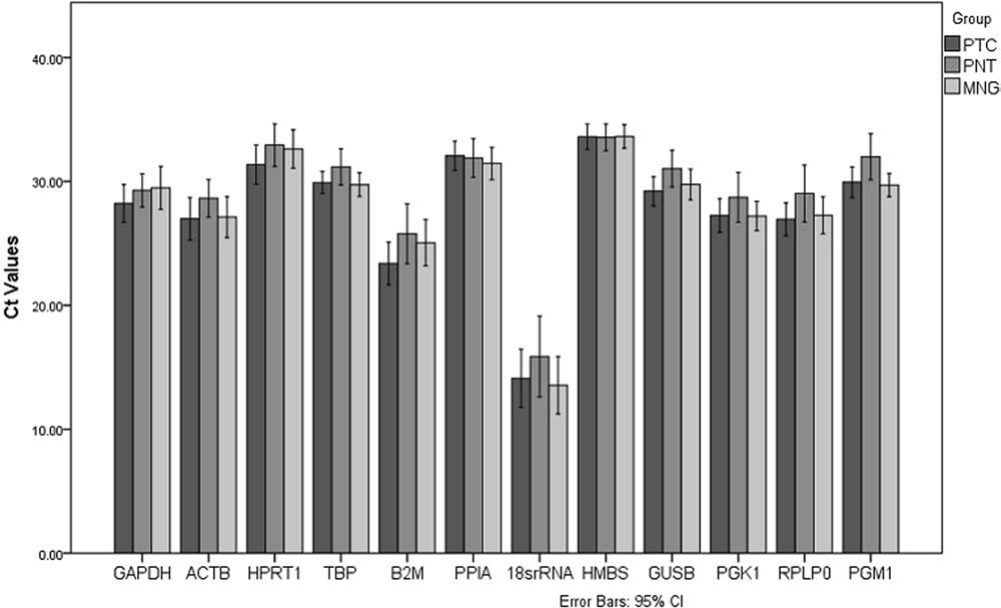
The mean Ct values and standard deviations of 12 candidate reference genes in papillary thyroid carcinoma (PTC), paired normal tissue (PNT) and multinodular goiter (MNG) groups.

Expression levels of the 12 reference genes in thyroid tissues are shown for PTC, PNT, and MNG groups in Fig. 1. For each HKG, the Ct coefficient of variation (CtCV%) value was calculated using the following formula: CtCV% = SD/mean × 100% (Table 2). According to these data, 18SrRNA showed the higher CtCV%, which means the greater level of dispersion around the mean. HMBS had the lower value of CtCv% which means the more precise estimation.

**Table 2:** The Ct coefficient of variation (CtCV%) values of 12 candidate reference genes in papillary thyroid carcinoma (PTC), paired normal tissue (PNT), multinodular goiter (MNG), and total tissues.

Gene	CtCV%			
	PTC	PNT	MNG	Total
GAPDH	9.7%	8.3%	10.6%	9.6%
ACTB	11.4%	9.6%	11.1%	10.8%
HPRT1	9.2%	9.4%	8.6%	9.1%
TBP	5.4%	8.5%	5.8%	6.9%
B2M	13.3%	17.0%	13.5%	15.0%
PPIA	6.6%	8.8%	7.5%	7.6%
18SrRNA	30.0%	37.2%	30.9%	33.4%
HMBS	5.5%	5.8%	5.1%	5.4%
GUSB	7.3%	8.6%	7.6%	8.1%
PGK1	9.0%	12.6%	7.9%	10.2%
RPLP0	8.9%	14.3%	9.9%	11.6%
PGM1	7.5%	10.5%	5.7%	8.8%

### Identification of the most stable reference genes

#### NormFinder algorithm

**Figure 2:**
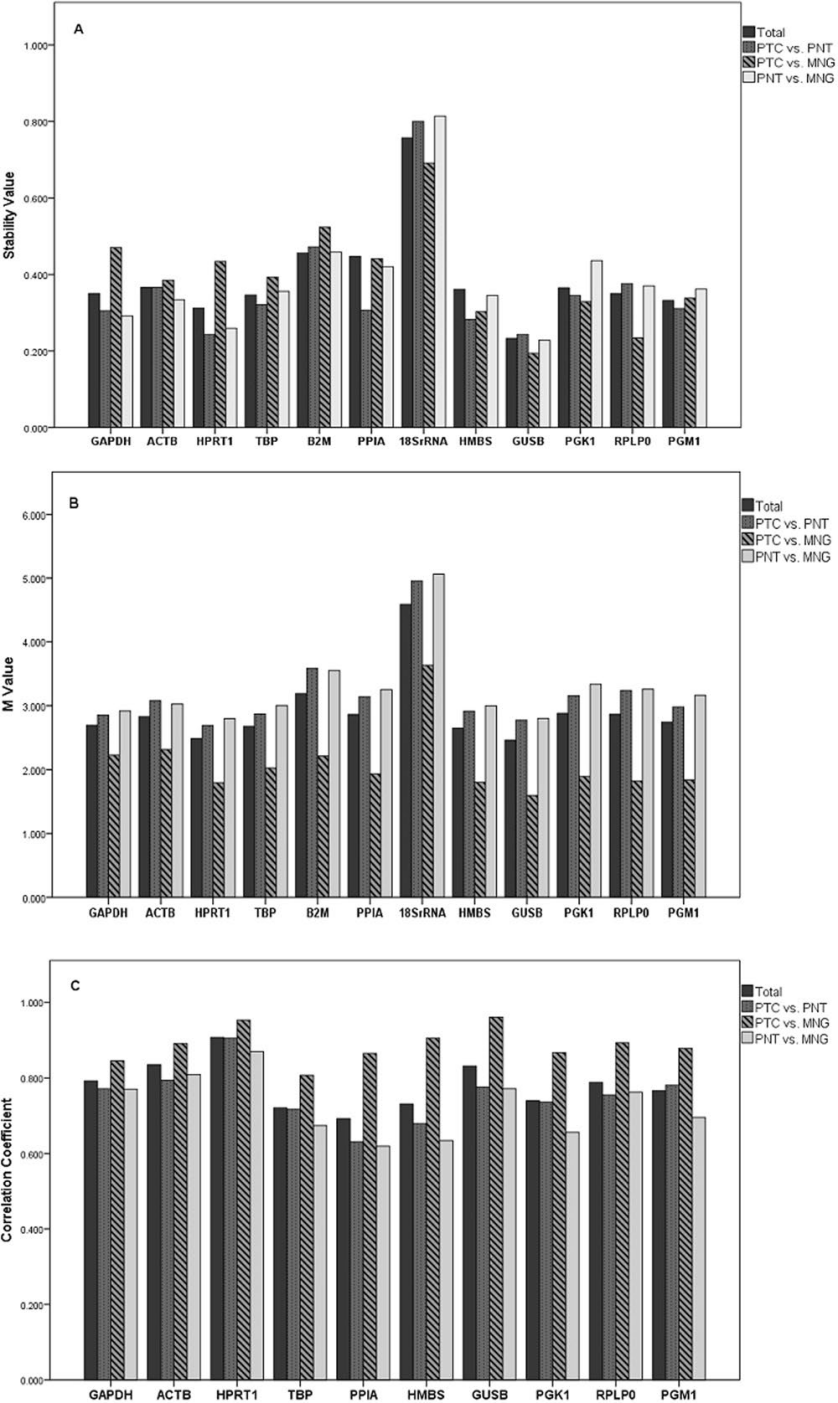
Candidate housekeeping gene’s stability results. (**A**) The NormFinder results. The lower stability value corresponds to more stably expressed gene. (**B**) The GeNorm results. The lower M-value corresponds to more stably expressed gene. (**C**) The BestKeeper results. The higher correlation coefficient corresponds to more stably expressed gene. PTC; papillary thyroid carcinoma, PNT; paired normal tissue, MNG; multinodular goiter.

This algorithm merges group division, the absolute copy number of the gene, and the random expression variations (stability value) caused by biological and experimental factors. Then, it ranks the reference genes in order; the lower the stability value the reliable the reference gene^8^. The five most stable HKGs in each studied group ranked by NormFinder (from most stable to least stable) were as follows: (i) Total: GUSB, HPRT1, PGM1, TBP, RPLP0; (ii) PTC vs. PNT: HPRT1, GUSB, HMBS, GAPDH, PPIA; (iii) PTC vs. MNG: GUSB, RPLP0, HMBS, PGK1, PGM1; (iv) PNT vs. MNG: GUSB, HPRT1, GAPDH, ACTB, HMBS (Fig. 2A). According to the NormFinder algorithm, the best combination of two HKGs are ‘GUSB and HPRT1’ or ‘GUSB and HMBS’ or ‘GUSB and PGM1’ (Table 3).

**Table 3:** The most stable housekeeping genes by NormFinder, GeNorm and BestKeeper.

Group	NormFinder		GeNorm	BestKeeper
	Single HKG	Combined HKGs	Single HKG	Single HKG
Total	GUSB	HMBS & GUSB	GUSB	HPRT1
PTC vs. PNT	HPRT1	HPRT1 & GUSB	HPRT1	HPRT1
PTC vs. MNG	GUSB	GUSB & PGM1	GUSB	GUSB
PNT vs. MNG	GUSB	HPRT1 & GUSB	HPRT1	HPRT1

HKG; housekeeping gene, MNG; multinodular goiter, PNT; paired normal tissue, PTC; papillary thyroid carcinoma.

#### GeNorm algorithm

**Figure 3:**
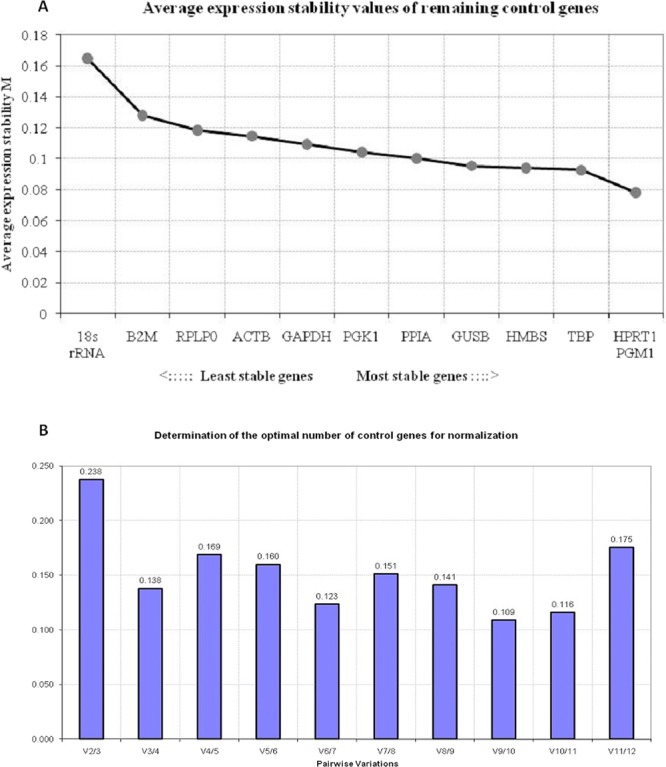
Selection of the most suitable reference genes using the GeNorm algorithm. (**A**) Average expression stability value (M) of housekeeping genes calculated by stepwise exclusion of the least stable genes. (**B**) Determination of the optimal number of housekeeping genes required for reliable normalization of qPCR data in thyroid tissues based on the pairwise variation values.

This algorithm calculates a stability value, M, as the average pair-wise variation of each reference gene in relation to all the other reference genes led to the elimination of the least stable gene. The process is followed by recalculation of the M values resulting in the ranking of the most stable genes. HKG with the lower M value has a higher expression stability^9^. Using GeNorm the five most stable HKGs evaluated (from most stable to least stable) were: (i) Total: GUSB, HPRT1, HMBS, TBP, GAPDH; (ii) PTC vs. PNT: HPRT1, GUSB, GAPDH, TBP, HMBS; (iii) PTC vs. MNG: GUSB, HPRT1, HMBS, RPLP0, PGM1; (iv) PNT vs. MNG: HPRT1, GUSB, GAPDH, HMBS, TBP (Fig. 2B). The GeNorm algorithm was also used to calculate the optimal number of reference genes required for a reliable normalization using a normalization factor (V_NF_ value). According to GeNorm, a combination of three reference genes was the optimal number of genes required for reliable normalization of qPCR data in thyroid tissues (Fig. 3).

#### BestKeeper algorithm

This algorithm computes the average Ct values, standard deviation (SD), and the coefficient of variation (CV) for each gene and SD > 1 is regarded as inappropriate for use as a stable reference gene. Then the program estimates the geometric mean of the stable control genes’ Ct values and creates pair-wise correlations between this index and each gene. HKG with the higher Pearson’s correlation coefficient value is considered as the most stable gene^10^. This tool has a limitation that it has been designed only for 10 genes, therefore, following the NormFinder and GeNorm analyses, the two least stable genes (B2M and 18SrRNA) were omitted then the algorithm was utilized. According to the BestKeeper, the five most stable HKGs in the groups studied (from most stable to least stable) were as follows: (i) Total: HPRT1, ACTB, GUSB, GAPDH, RPLP0; (ii) PTC vs. PNT: HPRT1, ACTB, PGM1, GUSB, GAPDH; (iii) PTC vs. MNG: GUSB, HPRT1, HMBS, RPLP0, ACTB; (iv) PNT vs. MNG: HPRT1, ACTB, GUSB, GAPDH, RPLP0 (Fig. 2C).

## Discussion

In order to monitor changes occurring in the pattern of PTC gene expression for the biomarkers identification, confirming stable and reliable internal reference genes for relative qRT-PCR is highly crucial. The ideal reference gene in thyroid tissue specimens should provide constant transcriptions at any types of thyroid tissue and at any time of the cell cycle or cellular differentiation. However, it is a desire and such as that HKG may probably not exist at all, so we can only find genes with the least expressed variation.

To select the suitable internal reference genes for relative quantification analysis in PTC, we chose a panel of 11 candidate HKGs by a systematic search then added PGM1 to the panel because of its stability according to our previous studies^11,12^. These genes were investigated in three types of thyroid lesion tissues including PTC, PNT, and MNG. The data obtained were analyzed by the three most commonly used software programs (NormFinder, GeNorm, and BestKeeper) for analyzing gene expression stability. Following the analysis, NormFinder and GeNorm selected the GUSB, and BestKeeper documented the HPRT1 as the most stable gene in the total sample group. All three algorithms recorded HPRT1 as the most stably expressed gene in PTC vs. PNT group. The three software recommended that GUSB is the most stable HKG in PTC vs. MNG. Also, NormFinder selected the GUSB, and GeNorm and BestKeeper selected the HPRT1 as the most stable gene in PNT vs. MNG group. Although, the three software programs use different mathematical models for the identification of the most stable reference gene, there were only minor variations in the selection of the single most stable reference gene by the three algorithms.

To date, only a few groups performed similar HKGs selection studies on human thyroid tissues or cells. In previous studies conducted by Weber *et al*. and Santin *et al*., ACTB was introduced as the best single reference gene among several other candidates tested^13,14^. Weber *et al*. evaluated 6 reference genes (ACTB, B2M, GAPDH, HPRT1, SDHA, and YWHAZ) in 14 thyroid specimens (7 normal thyroid tissues and 7 goitrous tissues) using the NormFinder algorithm^13^. Santin *et al*. evaluated 4 reference genes (ACTB, B2M, GAPDH, and TBP) in primary culture cells obtained from normal human thyroid tissue treated with progesterone or estradiol and chose NormFinder as an analyzer algorithm^14^. Contrary to these two studies, ACTB was ranked at the middle of the stable HKGs list in our study. The different results in studies are somewhat similar confirm that there is no universal or gold standard reference gene for all thyroid tissue samples because even highly stable expressed HKGs are prone to be affected by ethnicity and genetic status of examined tissues. On the other hand, diet, life style, and physical activity could have epigenetic effects influencing the expression of genes. In the case of cancerous tissues, due to the heterogeneity at the molecular, cellular and tissue levels, different results in similar studies may be obtained. However, pathological states of tissues and materials and methods used for an individual experiment are the other possible factors for inconsistency between our results and what the others have shown in previous studies. These different observations emphasize the need for determination of stably expressed reference genes in particular thyroid tissues and/or particular thyroid cell subtypes. Several studies on primary thyroid cancers and thyroid cancer-derived cell lines have employed GAPDH as a reliable internal reference gene because the transcription of GAPDH is stable in most experimental conditions^15–17^. However, the results of present investigation demonstrated GAPDH was not listed within the highest stable genes in the tissues and experimental setup used here. On the other hand, GAPDH has pseudogene, and it may amplify genomic DNA that lead to a false estimation of GAPDH expression^18^.

In our study conditions, using the three algorithms, GUSB and HPRT1 were identified as the most stably expressed genes in paired malignant and nonmalignant thyroid tissue samples. It is noteworthy that the results of the three software programs were close to each other. Therefore, since the rankings of the candidate HKGs were similar, our data suggest that GUSB and HPRT1 could be considered as the optimal internal reference genes for normalizing the relative gene quantitation in PTC studies. However, these findings are completely different from those found by Chantawibul *et al*. They demonstrated that GAPDH was the most and HPRT1 was the least stably expressed genes in thyroid tissues^19^. Although the stability value obtained with NormFinder was extraordinary high in Chantawibul *et al*. study, the differences may be attributed to the experimental factors and the sample selection included (13 goiters, 6 adenomas, 2 follicular carcinomas, 2 papillary carcinomas, and 2 lymphocytic thyroiditis).

At present, usage of the geometric mean of several reference genes rather than a single one is widely accepted to reduce the influence of fluctuations. Minimum Information for Publication of Quantitative Real-Time PCR Experiments (MIQE) recommends that the optimal number and the utility of HKGs must be validated for particular tissues and specific experimental conditions^20^. According to our study, GeNorm program suggested a combination of 3 most stable reference genes for gene expression studies in thyroid lesions. NormFinder software program found that the combination of GUSB and HMBS or HPRT1 or PGM1 were the best combinations.

In comparison to previous study, the results of current study provide promising evidence identifying potential HKGs for gene expression analyses in PTC. However, we stored the thyroid tissue samples in RNA later which is a commercial product and is not routinely used in investigations. Therefore, our results might not be similar to the samples were flash frozen and stored at −80 °C. For these types of stored thyroid tissues, the utility of reference genes must be validated. On the other hand, our study was limited to the most frequent type of thyroid cancer, PTC; however, there are several histological types and subtypes of thyroid cancer with different cellular origins, characteristics and prognoses including FTC and ATC (follicular thyroid cell-derived tumors), and MTC (parafollicular C cell-derived tumors). For these types of thyroid tissues, the stable reference genes must be investigated.

## Conclusions

GUSB and HPRT1 evidenced as the most stable reference genes when a panel of 12 candidate reference genes were compared in PTC, PNT, and MNG specimens stored in RNA later. These genes can be considered as single reference genes for normalization of relative qRT-PCR studies in PTC. However, it is suggested to utilize the validated combination of ‘GUSB and HPRT1’ or ‘GUSB and HMBS’ or ‘GUSB and PGM1’ as a much more reliable normalization strategy.

## Materials and Methods

### Literature review for selection of candidate reference genes

**Figure 4:**
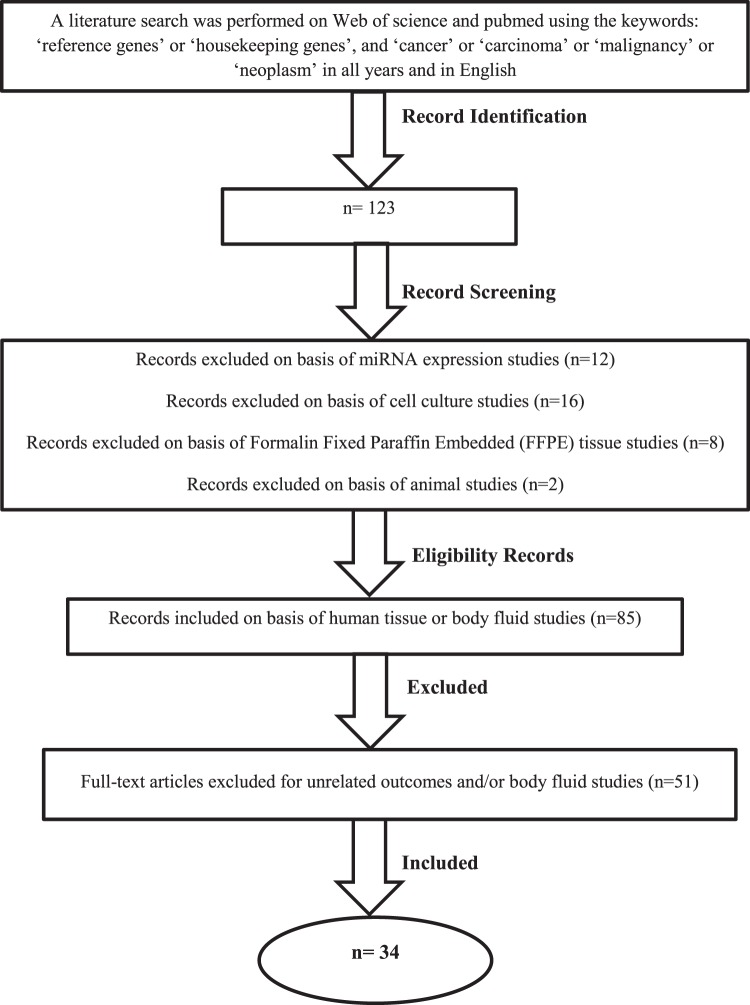
Review flow-chart for selection of included articles.

A systematic literature search was performed in Web of Science and PubMed databases using the following key words: ‘reference genes’ or ‘housekeeping genes’, and ‘cancer’ or ‘carcinoma’ or ‘malignancy’ or ‘neoplasm’ in English and in all years. The initial search visualized 123 articles. After applying the inclusion criteria (Fig. 4) 34 articles were included in the study^21–54^. According to the usage rate, 11 candidate reference genes were selected for validation in the study. The phosphoglucomutase 1 (PGM1) gene was also added to the list because of its stability that was exhibited in our previous RT-qPCR studies^11,12^. All selected reference genes are listed in Table 4.

**Table 4:** List of candidate reference genes used in the study.

Gene symbol		Gene ID	Official full name	Number of used/34 papers	% of used	Primer sequence (5′ → 3′)	Amplicon size (bp)
1	GAPDH	2597	glyceraldehyde-3-phosphate dehydrogenase	31	91.2	F: CTGCTCCTCCTGTTCGACAGT R: CCGTTGACTCCGACCTTCAC	100
2	ACTB	60	actin beta	29	85.3	F: GATCAAGATCATTGCTCCTCCT R: TACTCCTGCTTGCTGATCCA	108
3	HPRT1	3251	hypoxanthine phosphoribosyltransferase 1	27	79.4	F: CCCTGGCGTCGTGATTAGTG R: CACCCTTTCCAAATCCTCAGC	91
4	TBP	6908	TATA-box binding protein	23	67.6	F: CACGAACCACGGCACTGATT R: TTTTCTTGCTGCCAGTCTGGAC	89
5	B2M	567	beta-2-microglobulin	22	64.7	F: TGCCTGCCGTGTGAACCATG R: TGCGGCATCTTCAAACCTCCA	97
6	PPIA	5478	peptidylprolyl isomerase A	18	52.9	F: GCTGTGAGGAGGTACTGCTTG R: CCTGAGAAACCAAGTCCTTAGTG	145
7	18SrRNA	100008588	RNA, 18S ribosomal N5	17	50	F: GGCCCTGTAATTGGAATGAGTC R: CCAAGATCCAACTACGAGCTT	146
8	HMBS	3145	hydroxymethylbilane synthase	16	47.1	F: GCACCCACACACAGCCTACT R: GTACCCACGCGAATCACTCTC	108
9	GUSB	2990	glucuronidase beta	13	38.2	F: CGCCCTGCCTATCTGTATTC R: TCCCCACAGGGAGTGTGTAG	91
10	PGK1	5230	phosphoglycerate kinase 1	11	32.4	F: AGCGGGTCGTTATGAGAGTC R: TGGTACAGCAGCCTTAATCC	86
11	RPLP0	6175	ribosomal protein lateral stalk subunit P0	11	32.4	F: CCTCGTGGAAGTGACATCGT R: CTGTCTTCCCTGGGCATCAC	76
12	PGM1	5236	phosphoglucomutase 1	—	—	F: AGCATTCCGTATTTCCAGCAG R: GCCAGTTGGGGTCTCATACAAA	120

The eleven most widely used reference genes in tissue cancer studies are ranked from 1 to 11.

### Tissue samples

Patients who had undergone near-total or total thyroidectomy from November 2015 to August 2016 in Shariati Hospital, Tehran Iran were initially enrolled in the study. The present study was approved by the Institutional Review Board and Ethics Committee of Research Institute for Endocrine Sciences, Shahid Beheshti University of Medical Sciences, Tehran, Iran (Ethical approval number: IR.SBMU.REC.1397.110) and written informed consent was obtained from all participants.

Following the thyroid excision, part of the tumor and adjacent tissues were immediately treated in RNAlater® RNA Stabilization Reagent (Qiagen, Hilden, Germany) prior to further processing. Before archival storage at −80 °C, the tissues were incubated overnight in RNAlater at 2–8 °C, and then they were removed from the reagent and transferred to −80 °C until tested.

According to the postoperative pathological reports and the histological confirmation, a total of 45 thyroid tissue samples taken from 30 thyroid gland consisting of 15 PTC, 15 PNT belonged to the patients with PTC tumors, and 15 goiterus tissues belonged to the patients with MNG were selected. The approximate size of each sample was 0.5 cm. The pathological characteristics of the tissues used in the study are summarized in Table 5. Tumor staging was determined by the 7th edition of the American Joint Committee on Cancer Tumor-Node-Metastasis (TNM) staging system^55^.

**Table 5:** Pathologic characteristics of the study tissues.

Parameter	PTC	PNT	MNG	Total
Tissue number	15	15	15	45
**Histopathology**				
multinodular goiter			15	
classic PTC	14	—		—
FVPTC	1			
**Tumor size (cm)**				
≤2	9	—	—	—
2–4	6			
**Invasion**				
Negative	10	—	—	—
Positive^a^	5			
**Lymph node metastasis** ^**b**^				
Negative	7	—	—	—
Positive	8			
**TNM stage** ^**c**^				
I, II	10	—	—	—
III, IV	5			

FVPTC; follicular variant of papillary thyroid carcinoma, MNG; multinodular goiter, PNT; paired normal tissue, PTC; papillary thyroid carcinoma.

^a^Includes 4 extracapsular invasion, and 1 extracapsular and lymphovascular invasions.

^b^Includes N1a and/or N1b.

^c^American Joint Committee on Cancer (AJCC) Tumor-Node-Metastasis (TNM) staging system.

### RNA extraction

Total RNA was extracted from each tissue sample using TRIzol reagent (Invitrogen Life Technologies, Carlsbad, CA, USA), according to the manufacturer’s instructions.

DNase I was used to eliminate residual genomic DNA contamination. The quantity and quality of the isolated RNA were measured using NanoDropND-1000 spectrophotometer (ThermoScientific, Waltham, MA, USA). The purity of total RNA was determined using the A260/A280 ratio. The integrity of the RNA samples was confirmed by electrophoresis on 1.0% agarose gel (Ultra-Pure™ Agarose; Invitrogen).

### Complementary DNA (cDNA) synthesis

Three µg of total RNA was reversely transcribed to cDNA using Thermo Scientific RevertAid Reverse Transcriptase (1 µL of 200 U/µL), random hexamer primers (1 µl of 100 µM), dNTPS (2 µL of 10 mM), and RiboLock RNase-inhibitor (0.5 µL of 40 U/µL), incubated for 10 min at 25 °C, followed by 60 min at 42 °C in a total volume of 20 µL. The reaction was terminated by heating the reactions at 70 °C for 10 min.

### Quantitative polymerase chain reaction (qPCR)

Primer sequences of 11 candidate reference genes were designed using the Primer 3 and Gene Runner software. Primer sequences of PGM1 were taken from the previously published study^11^ (Table 4). qPCRs were performed using the rotor gene 6000 real-time PCR machine (Corbett, Life science, Sydney, Australia). All reactions were set up for total volumes of 10 µL which contained 1 µL cDNA, 0.5 µL of each forward and reverse primer, 3.75 µL of SYBR Green PCR Master Mix 2X (ThermoFisher, USA), and 4.25 µL of nuclease-free water. The cycling profile used for PCR reactions was as follows: 10 min at 95 °C for initial denaturation, followed by 40 cycles with 15 s at 95 °C, 45 s at 60 °C and 40 s at 72 °C. All samples were run in triplicate and no cDNA template samples were used as negative controls.

The specificity of the real-time PCR reactions was verified by the melt curves analysis. For the PCRs efficiency investigation, several cDNA samples were selected randomly and were diluted with a 10-fold serial dilution ranging from 1X to 100,000X. Slope, intercept, Pearson’s correlation coefficient, coefficient of determination, and amplification efficiency were calculated by the Rotor-Gene 6000 Series Software (Table 1).

### Statistical analysis

NormFinder^8^, GeNorm^9^, and BestKeeper^10^ statistical algorithm, the three frequently used software programs, were applied to determine the most stable reference genes. For further statistical data analysis, SPSS 20.0 software (Chicago, IL, USA) was used.

### Ethical approval

The study was approved by the Institutional Review Board and Ethics Committee of Research Institute for Endocrine Sciences, Shahid Beheshti University of Medical Sciences, Tehran, Iran (Ethical approval number: IR.SBMU.REC.1397.110). All procedures performed in the study involving human participants were in accordance with the 1964 Helsinki declaration and its later amendments or comparable ethical standards.

## Supplementary information

Supplemental Figures
