# Supplementary material for: Validation of Reference Genes for Normalization of Relative qRT-PCR Studies in Papillary Thyroid Carcinoma
Source: Sci Rep. 2019 Oct 23;9:15241. doi: 10.1038/s41598-019-49247-1 (PMC6811563; doi:10.1038/s41598-019-49247-1)

Validation of Reference Genes for Normalization of Relative qRT-PCR Studies in Papillary Thyroid Carcinoma

S. Adeleh Razavi[1,2,3], Mandana Afsharpad[4], Mohammad Hossein Modarressi[5], Maryam Zarkesh[1], Parichehreh Yaghmaei[3], Shirzad Nasiri[6], S. Mohammad Tavangar[7], Hanieh Gholami[8], Afsoon Daneshafrooz[1], Mehdi Hedayati[1*]

1) Cellular and Molecular Endocrine Research Center, Research Institute for Endocrine Sciences, Shahid Beheshti University of Medical Sciences, Tehran, Iran
2) Department of Research and Development (R&D), Saeed Pathobiology & Genetics Laboratory, Tehran, Iran
3) Department of Biology, Faculty of Basic Sciences, Science and Research Branch, Islamic Azad University, Tehran, Iran
4) Cancer Control Research Center, Cancer Control Foundation, Iran University of Medical Sciences, Tehran, Iran
5) Department of Medical Genetics, School of Medicine, Tehran University of Medical Sciences, Tehran, Iran
6) Surgery Department, Shariati Hospital, Tehran University of Medical Sciences, Tehran, Iran
7) Department of Pathology, Shariati Hospital, School of Medicine, Tehran University of Medical Sciences, Tehran, Iran
8) Endocrine Physiology Research Center, Research Institute for Endocrine Sciences, Shahid Beheshti University of Medical Sciences, Tehran, Iran

Corresponding author: Mehdi Hedayati

Supplemental Fig. 1. Standard curves of all included reference genes

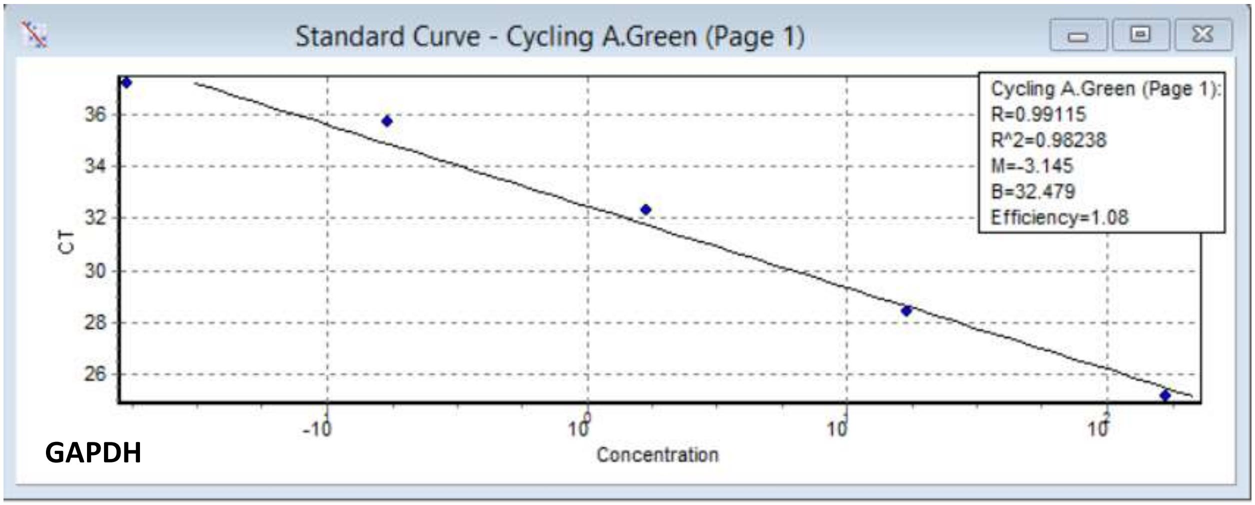

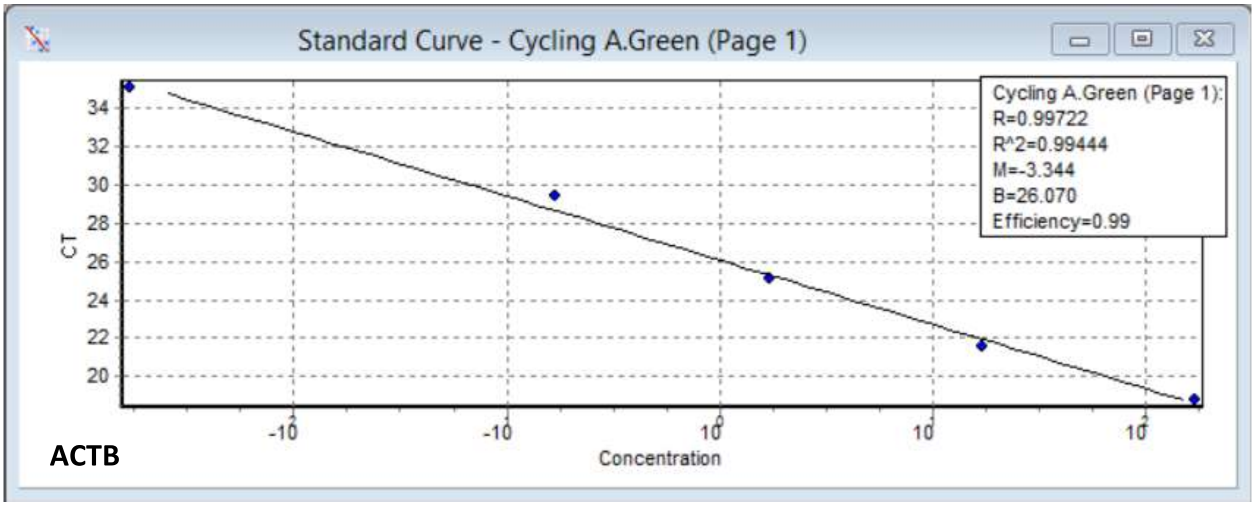

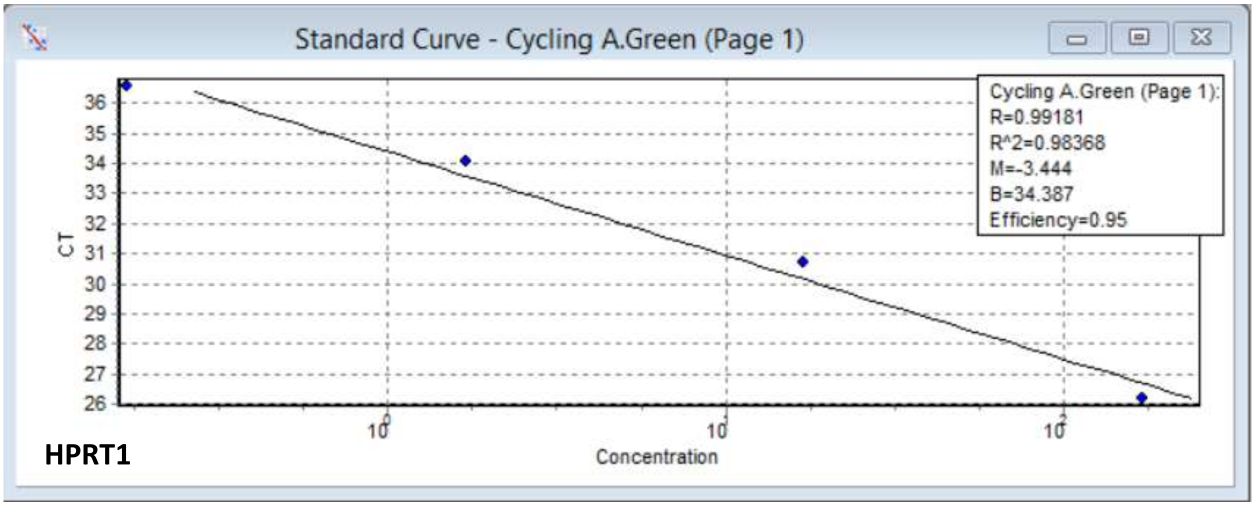

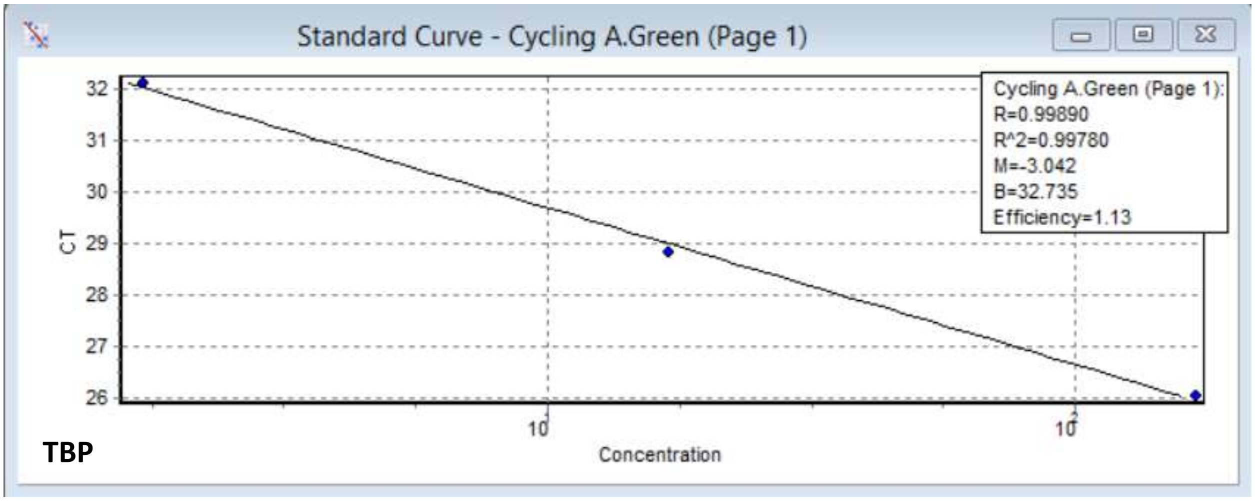
Standard Curve - Cycling A.Green (Page 1)
Cycling A.Green (Page 1):
R=0.99890
R^2=0.99780
M=-3.042
B=32.735
Efficiency=1.13
CT
Concentration

TBP

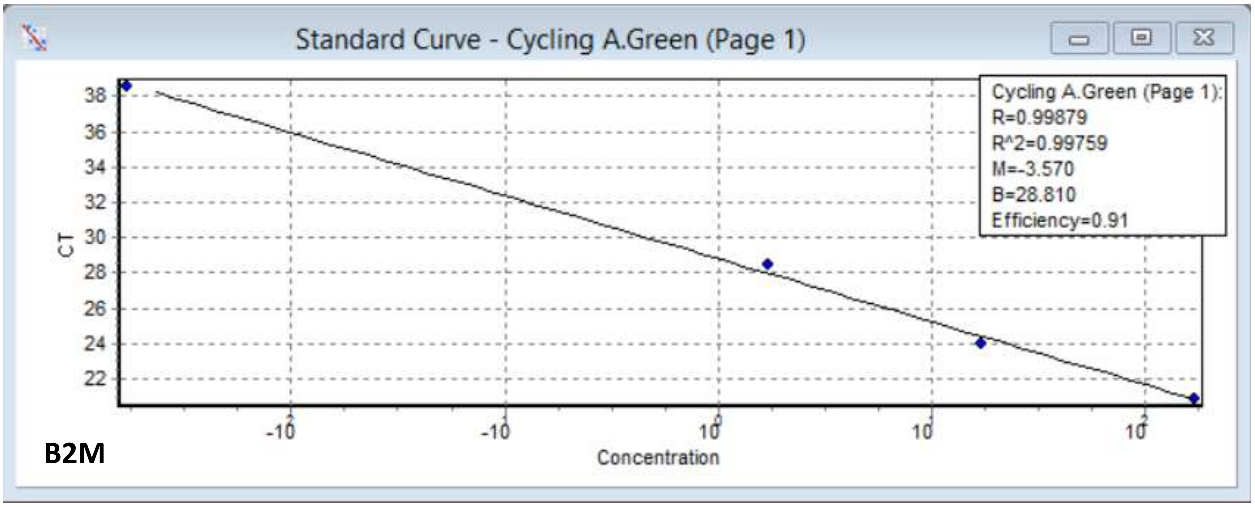
Standard Curve - Cycling A.Green (Page 1)
Cycling A.Green (Page 1):
R=0.99879
R^2=0.99759
M=-3.570
B=28.810
Efficiency=0.91
CT
Concentration

B2M

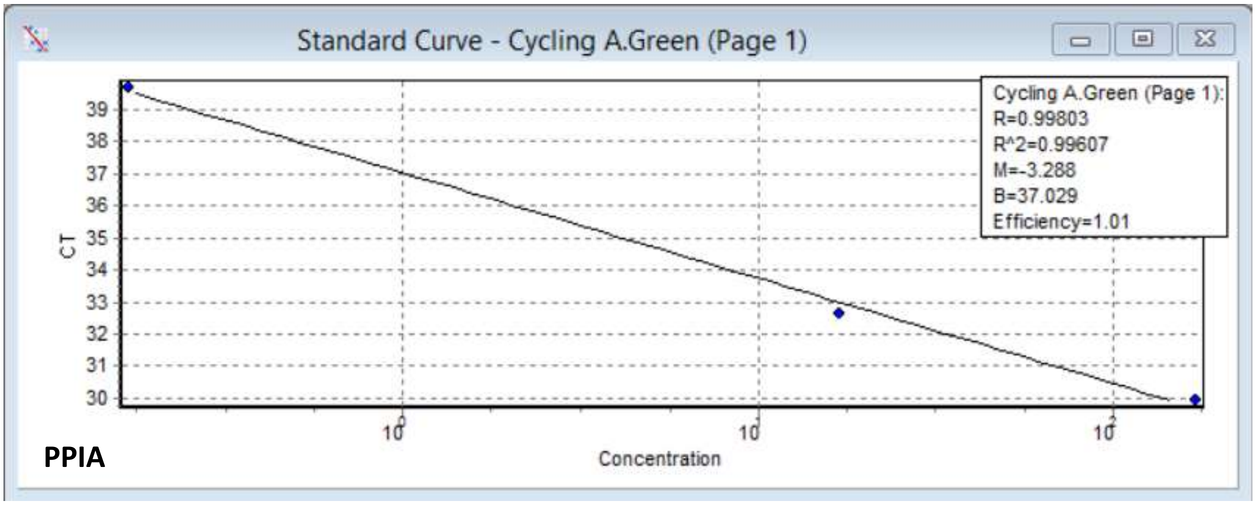
Standard Curve - Cycling A.Green (Page 1)
Cycling A.Green (Page 1):
R=0.99803
R^2=0.99607
M=-3.288
B=37.029
Efficiency=1.01
CT
Concentration

PPIA

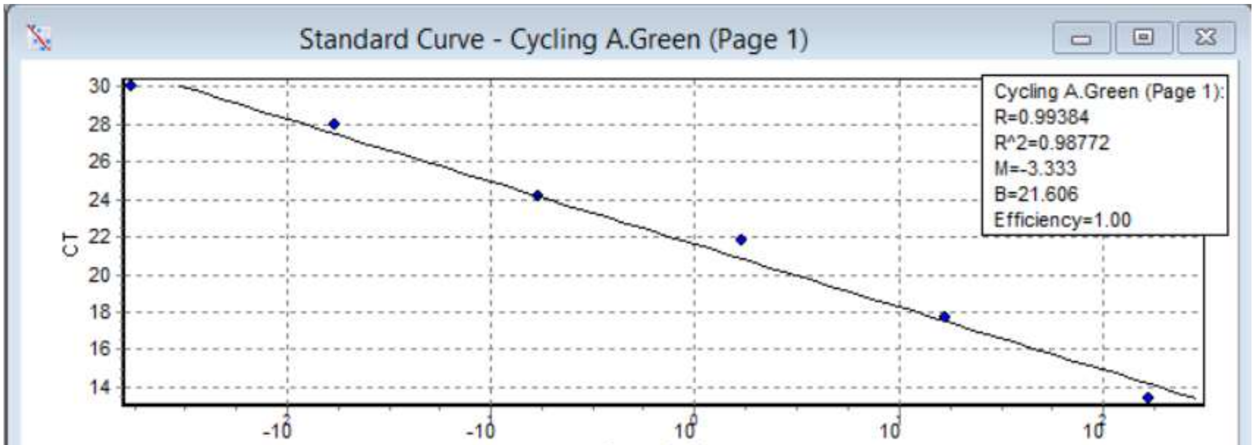

18SrRNA

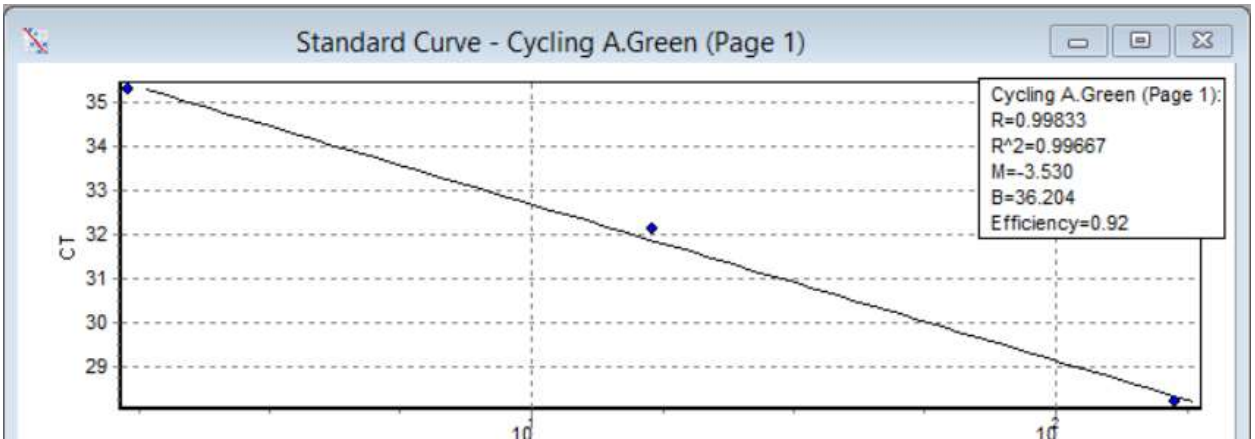

HMBS

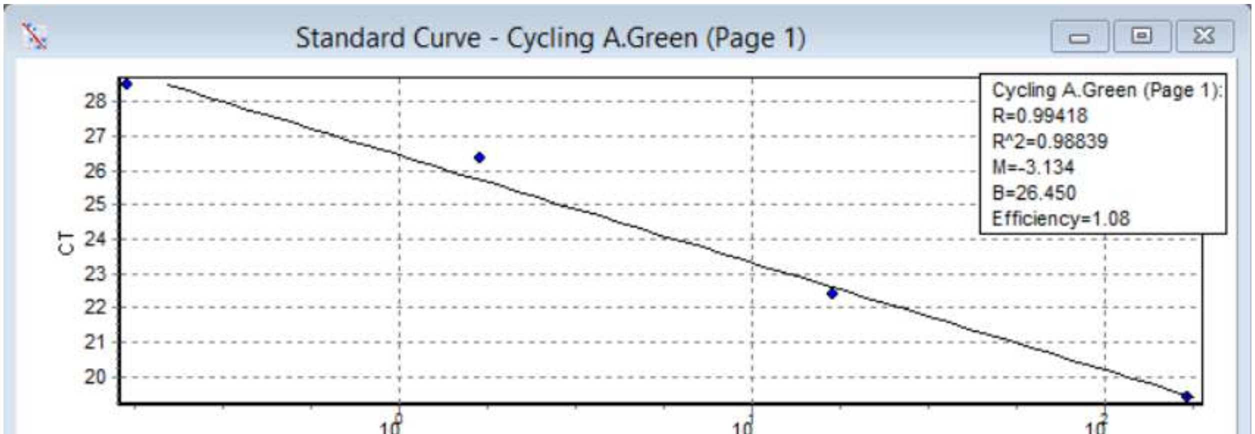

GUSB

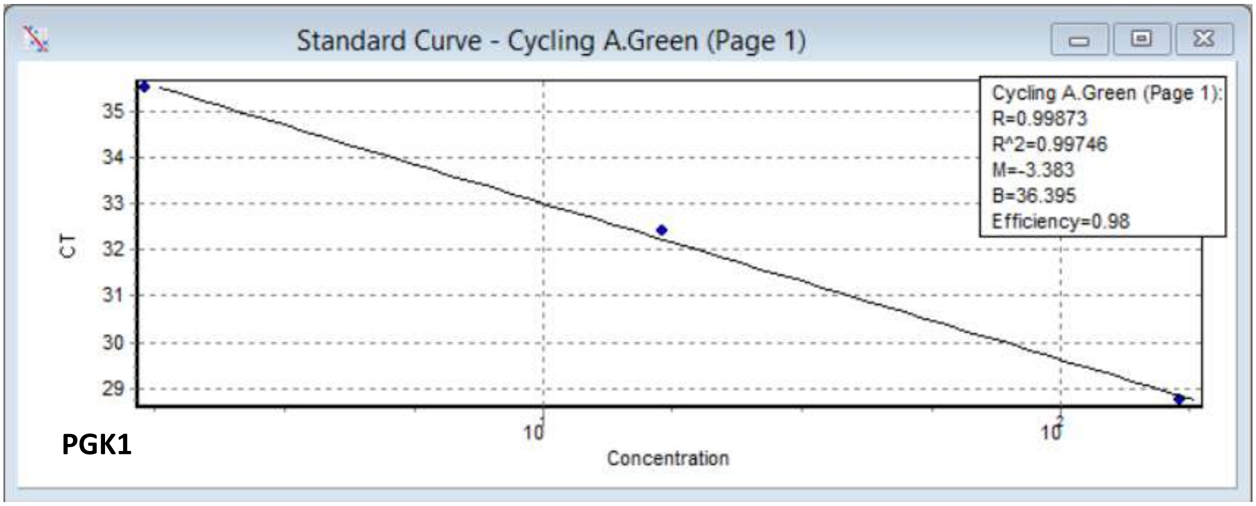
Standard Curve - Cycling A.Green (Page 1)
Cycling A.Green (Page 1):
R=0.99873
R^2=0.99746
M=-3.383
B=36.395
Efficiency=0.98
CT
35
34
33
32
31
30
29
10
10
Concentration
PGK1

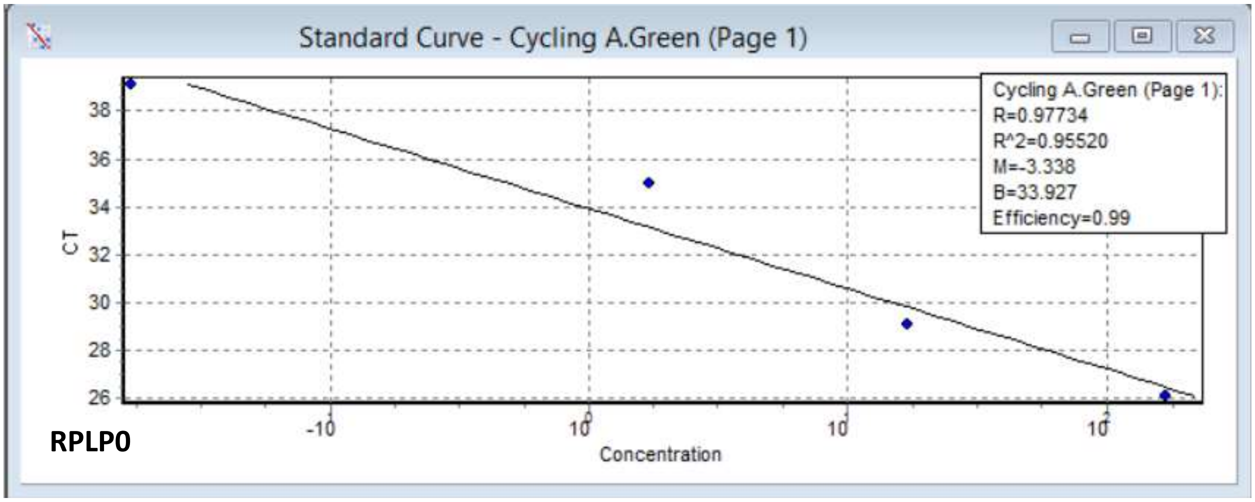
Standard Curve - Cycling A.Green (Page 1)
Cycling A.Green (Page 1):
R=0.97734
R^2=0.95520
M=-3.338
B=33.927
Efficiency=0.99
CT
38
36
34
32
30
28
26
-10
10
10
10
Concentration
RPLP0

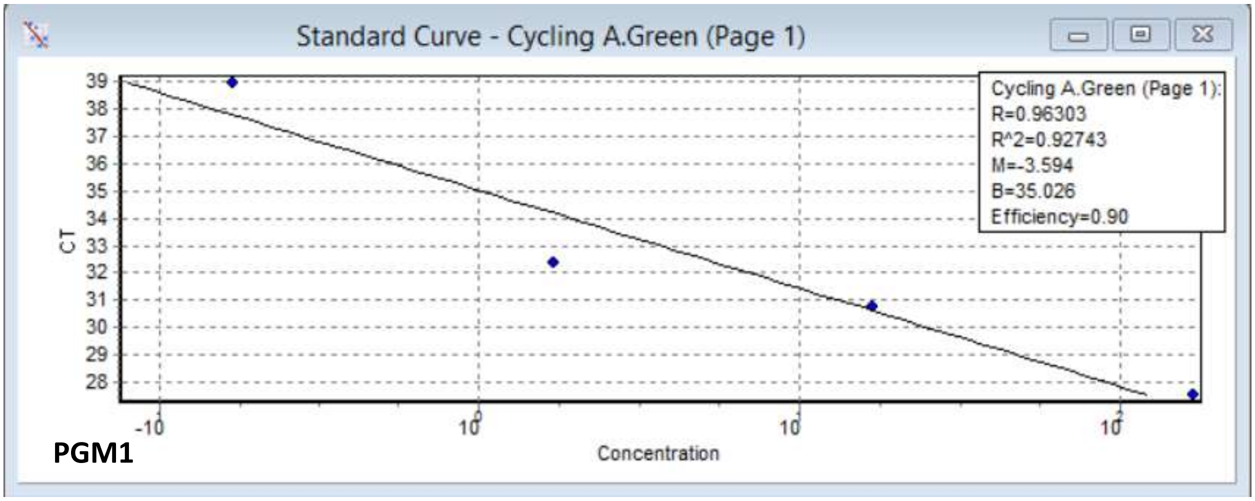
Standard Curve - Cycling A.Green (Page 1)
Cycling A.Green (Page 1):
R=0.96303
R^2=0.92743
M=-3.594
B=35.026
Efficiency=0.90
CT
39
38
37
36
35
34
33
32
31
30
29
28
-10
10
10
10
Concentration
PGM1

Supplemental Fig. 2. Melt curves and amplification plots of all included reference genes

GAPDH

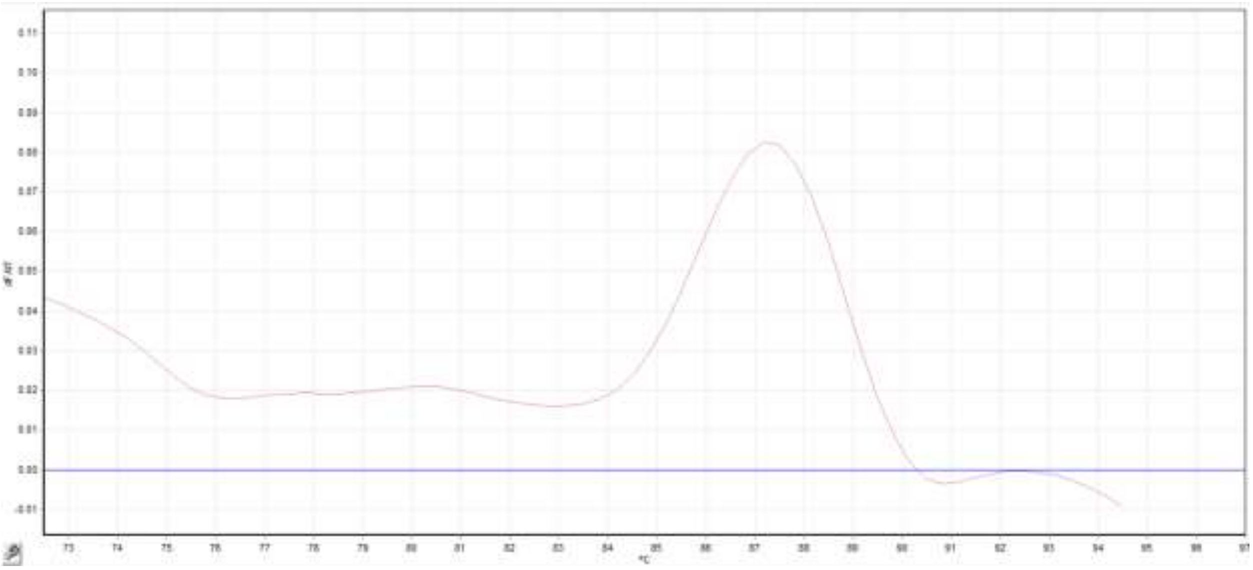

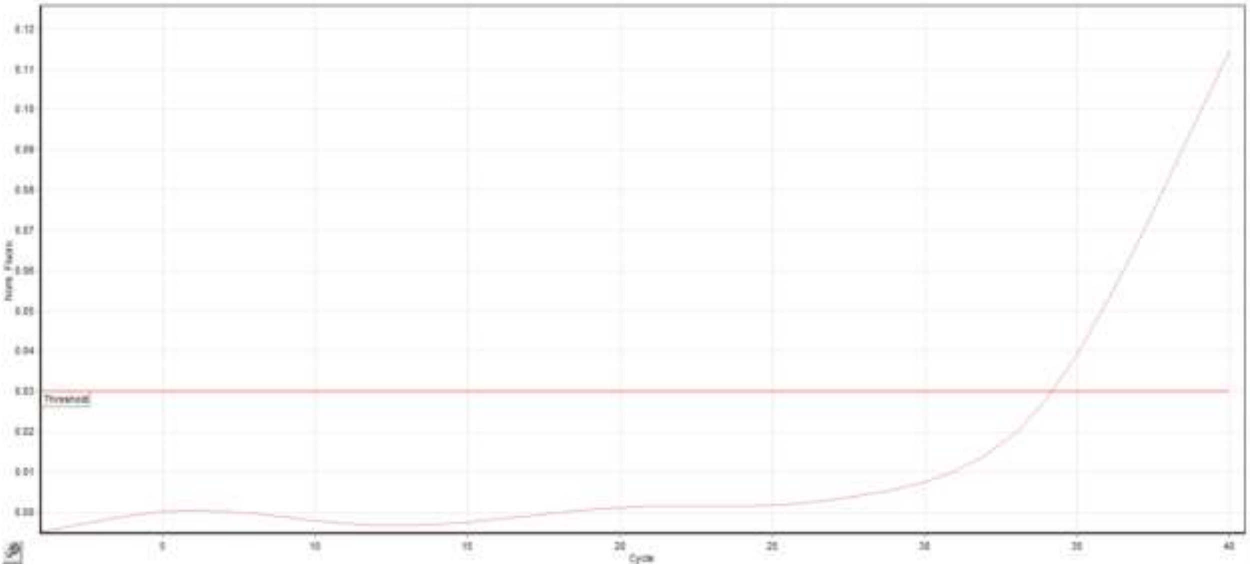

ACTB

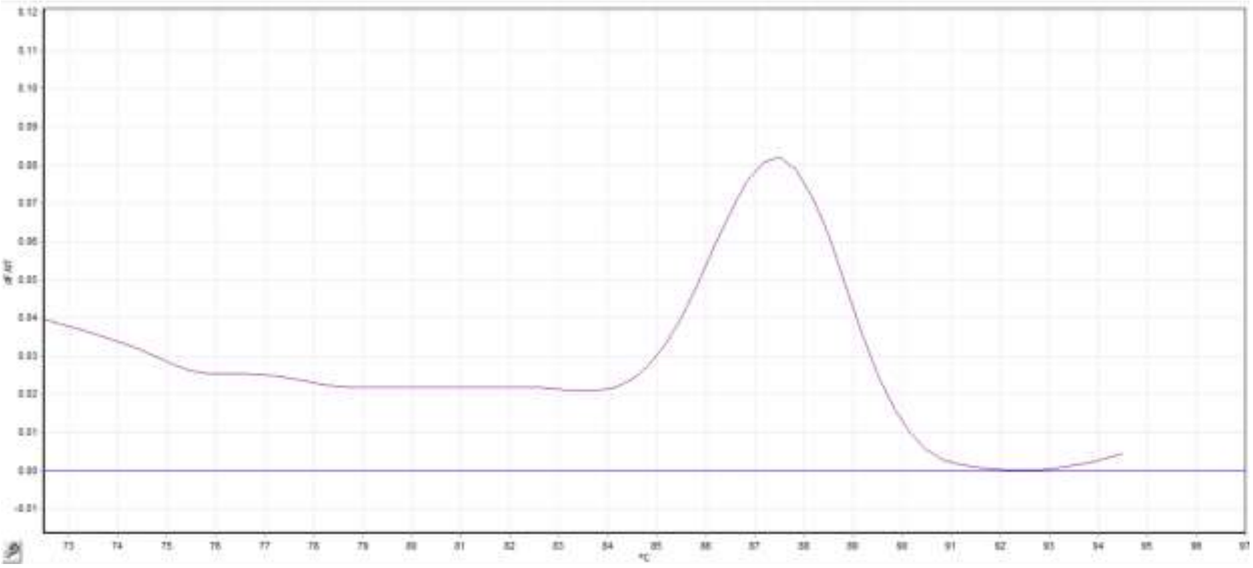

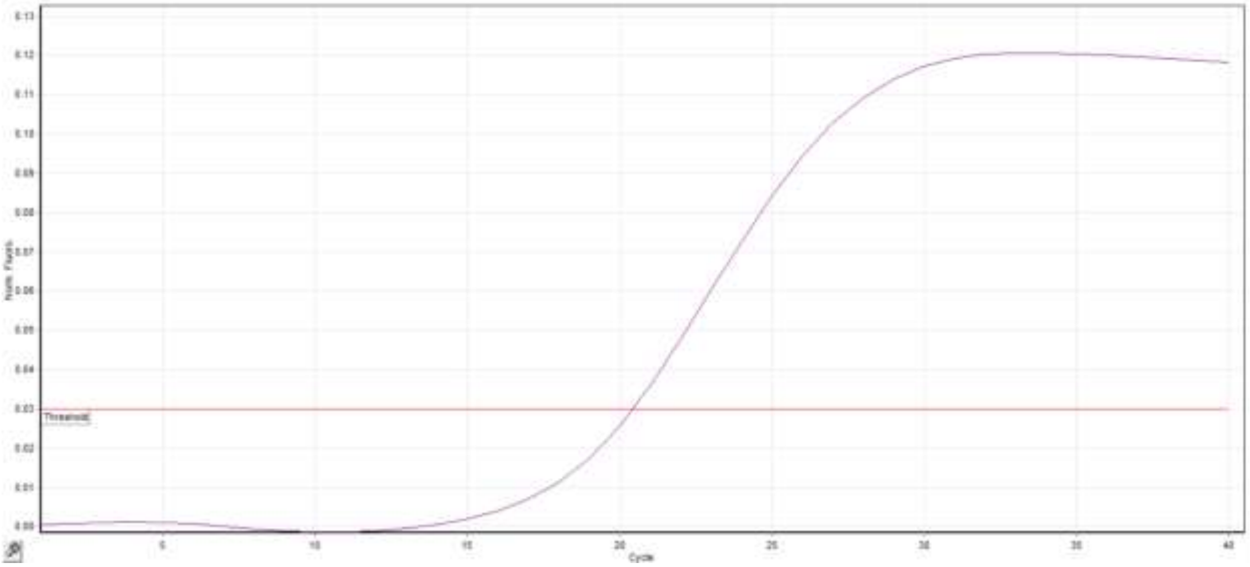

HPRT1

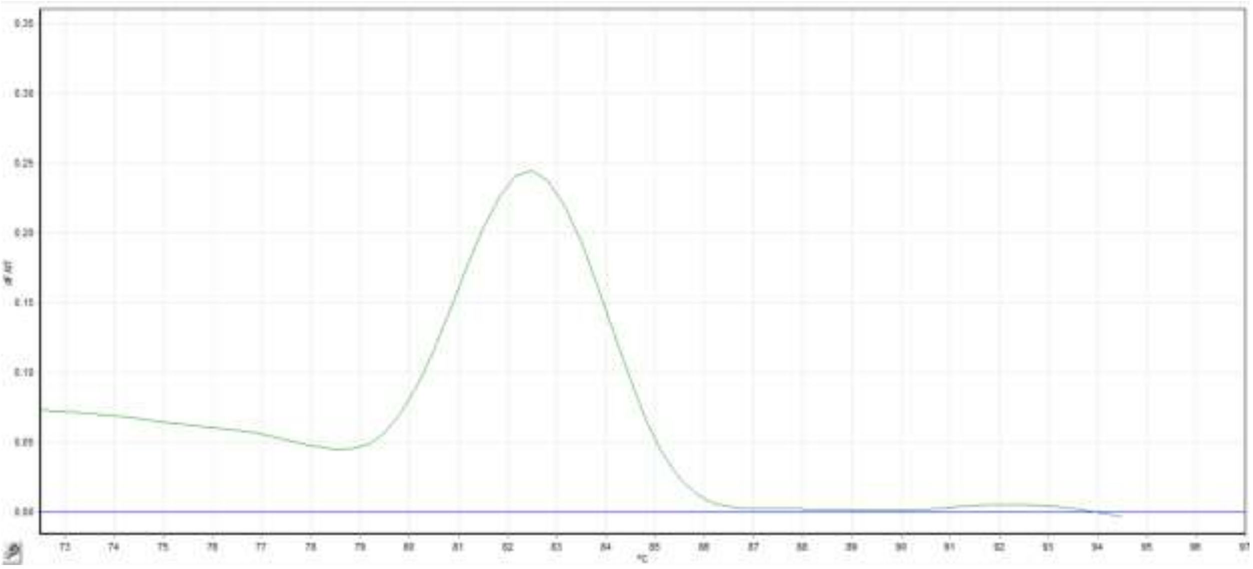

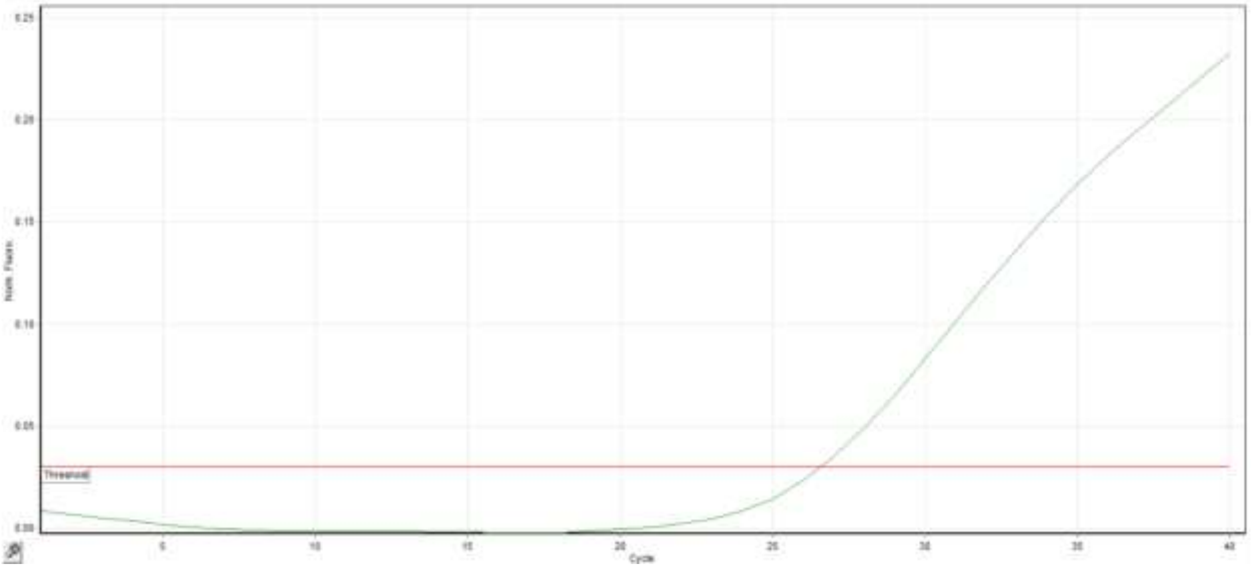

TBP

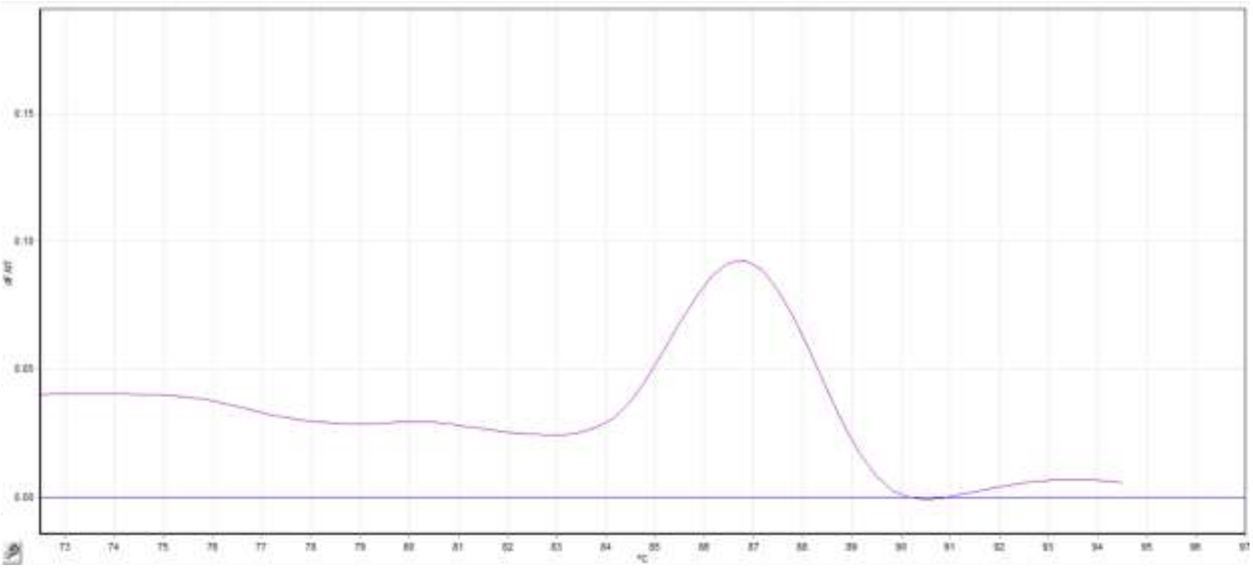

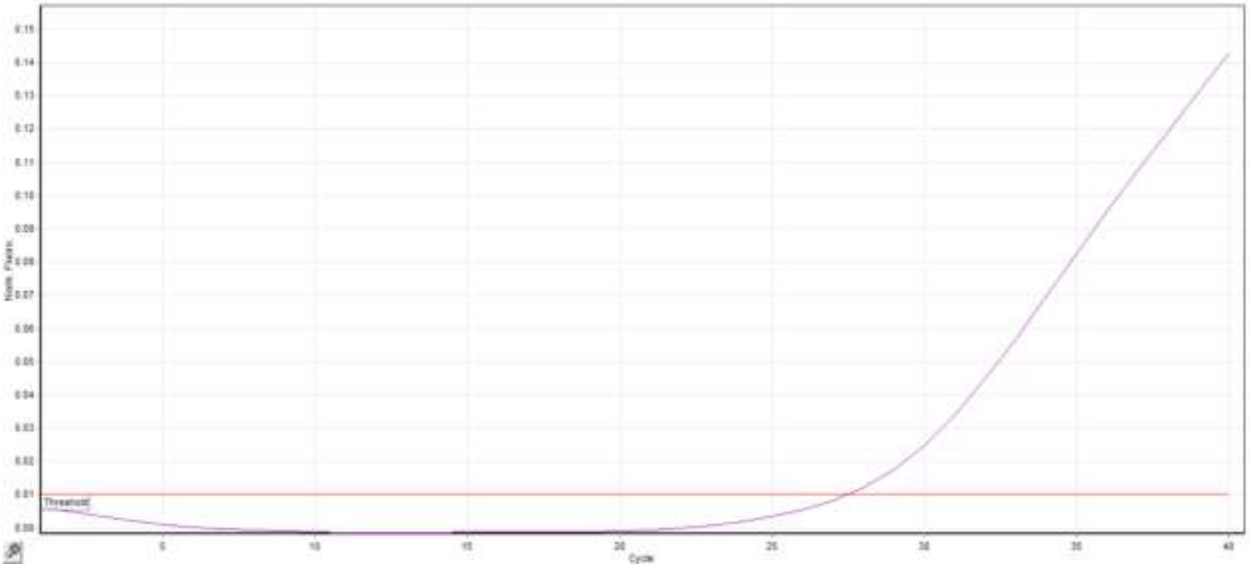

B2M

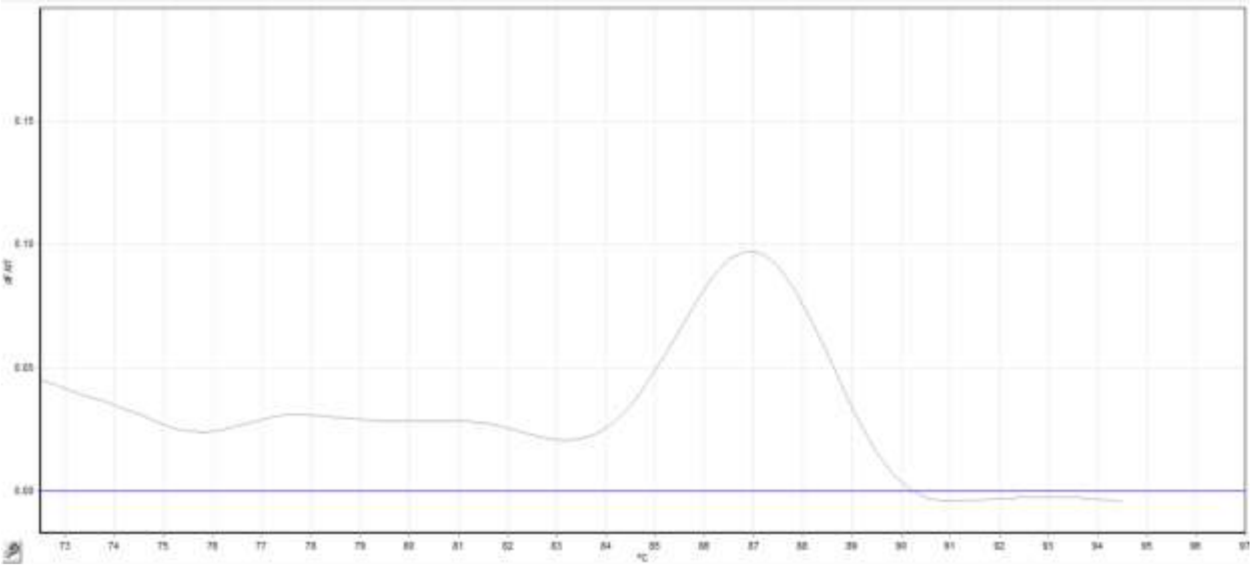

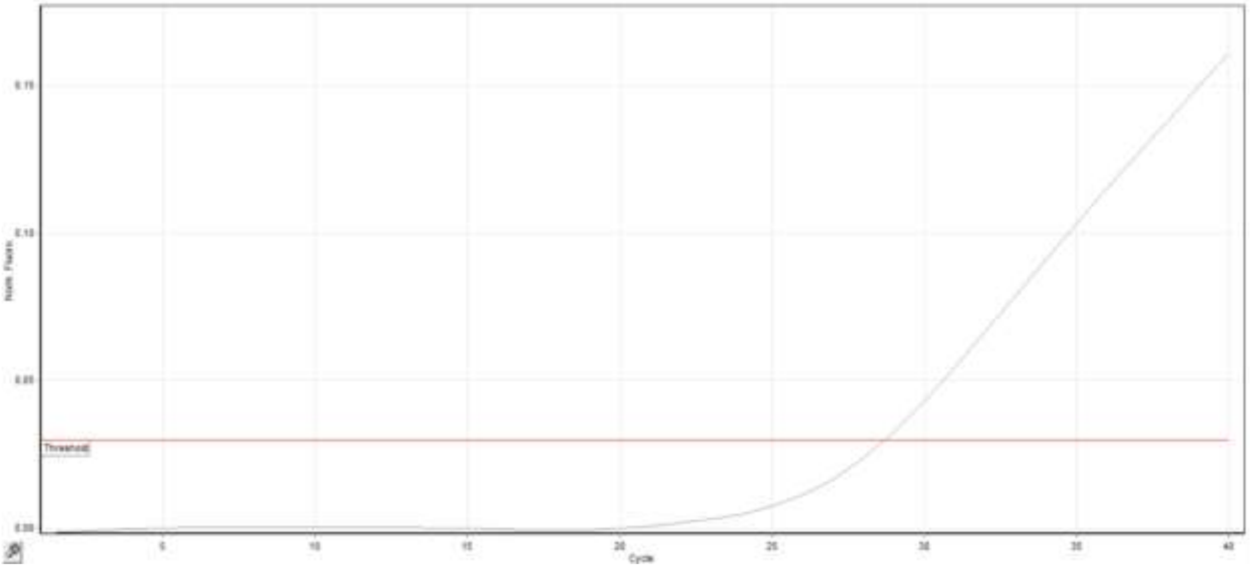

PPIA

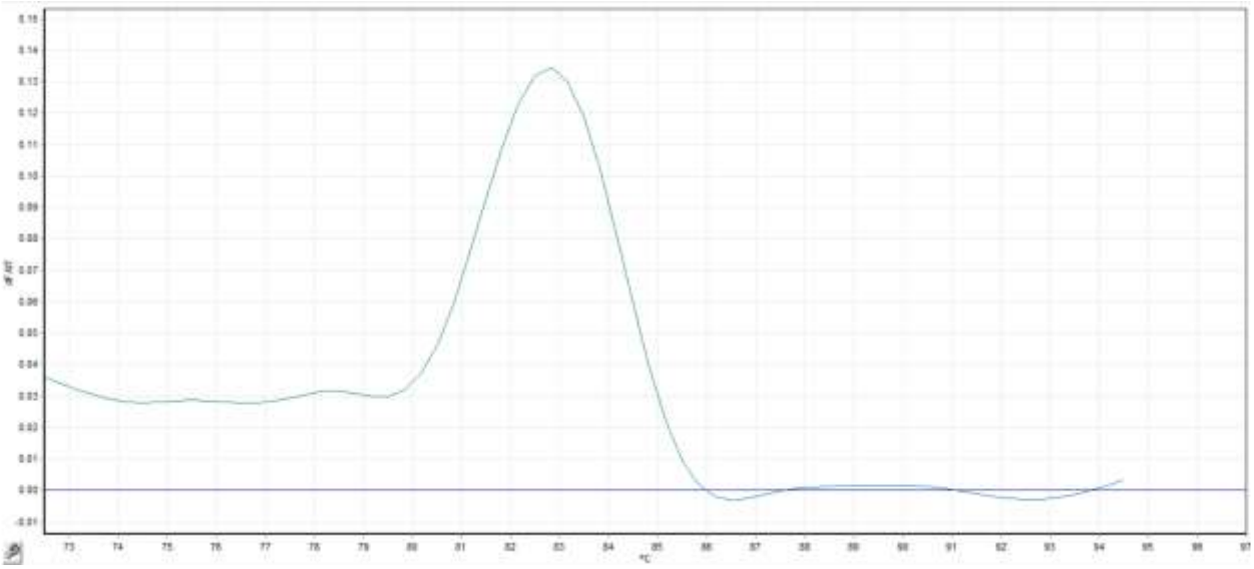

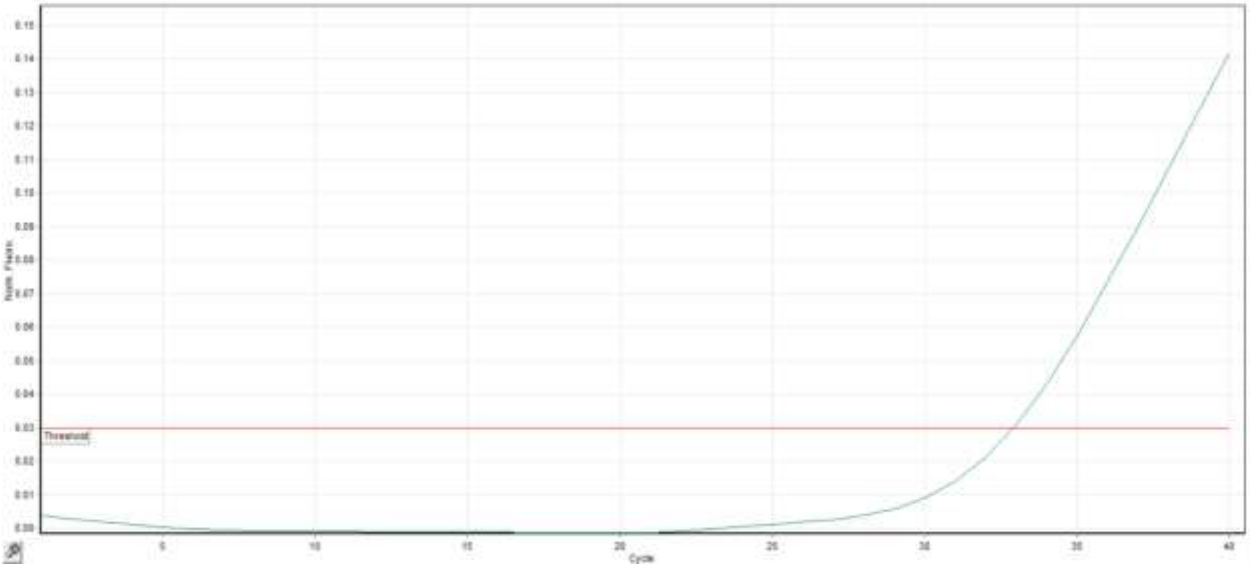

18SrRNA

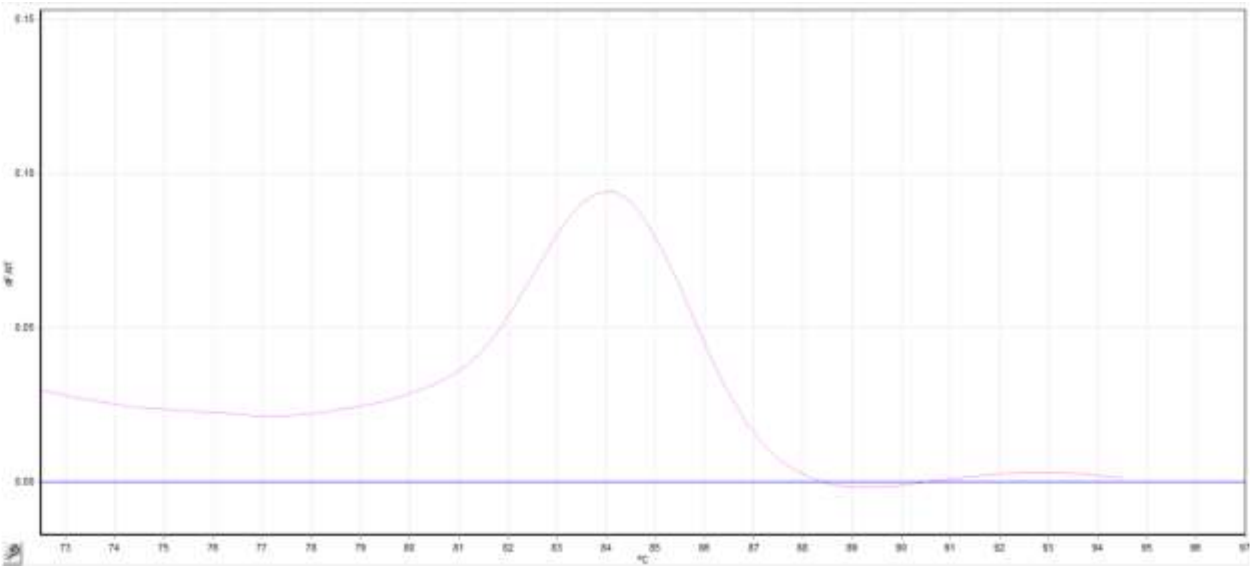

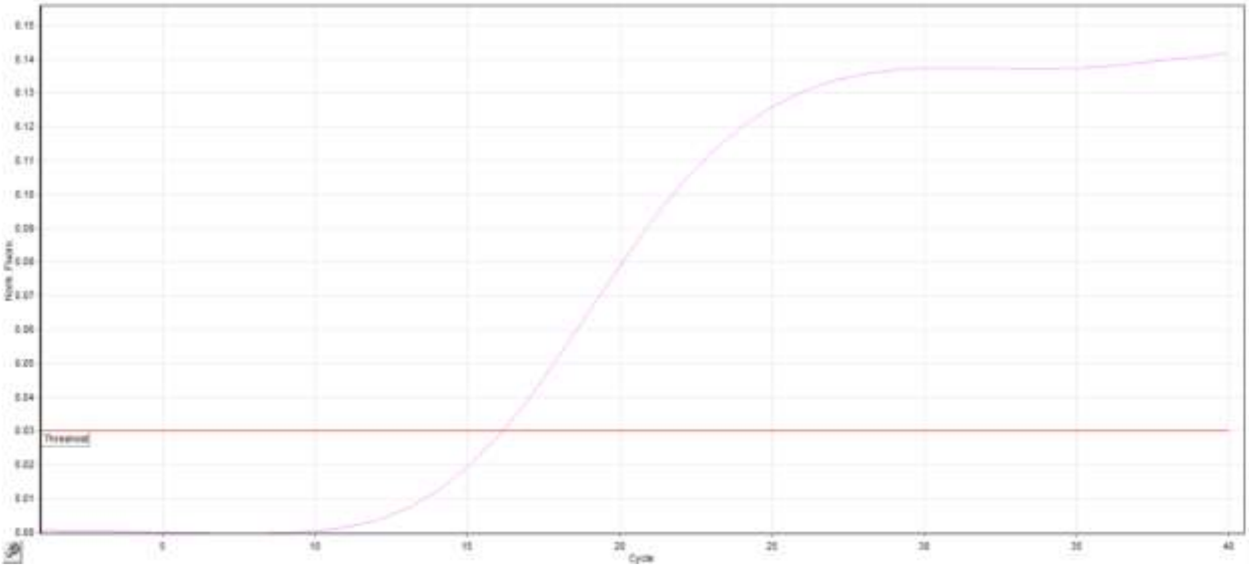

HMBS

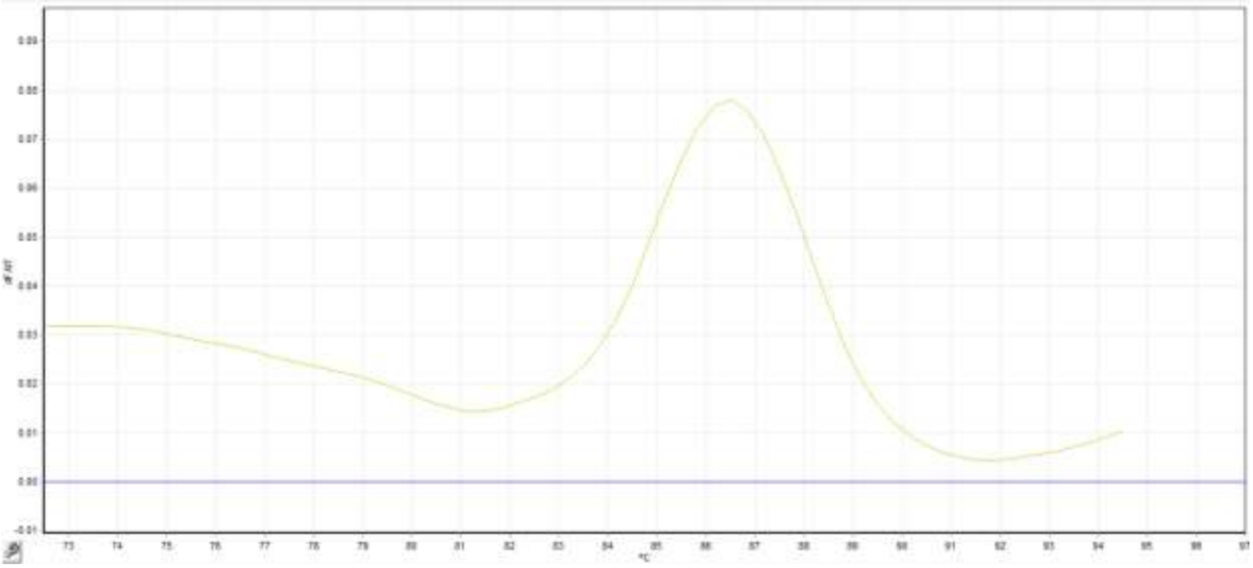

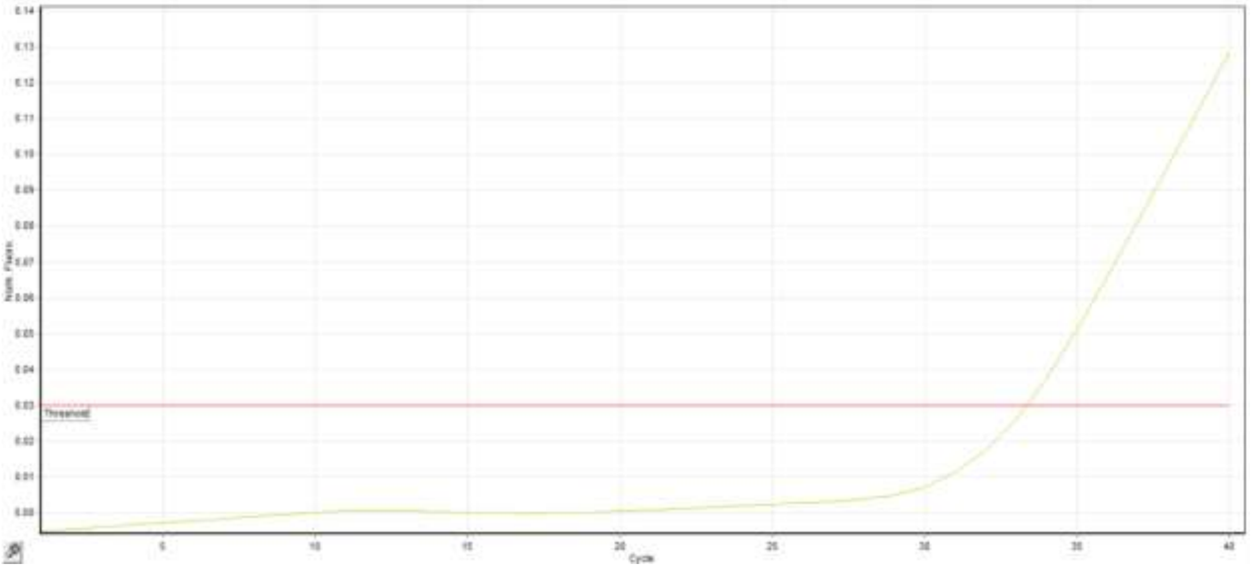

GUSB

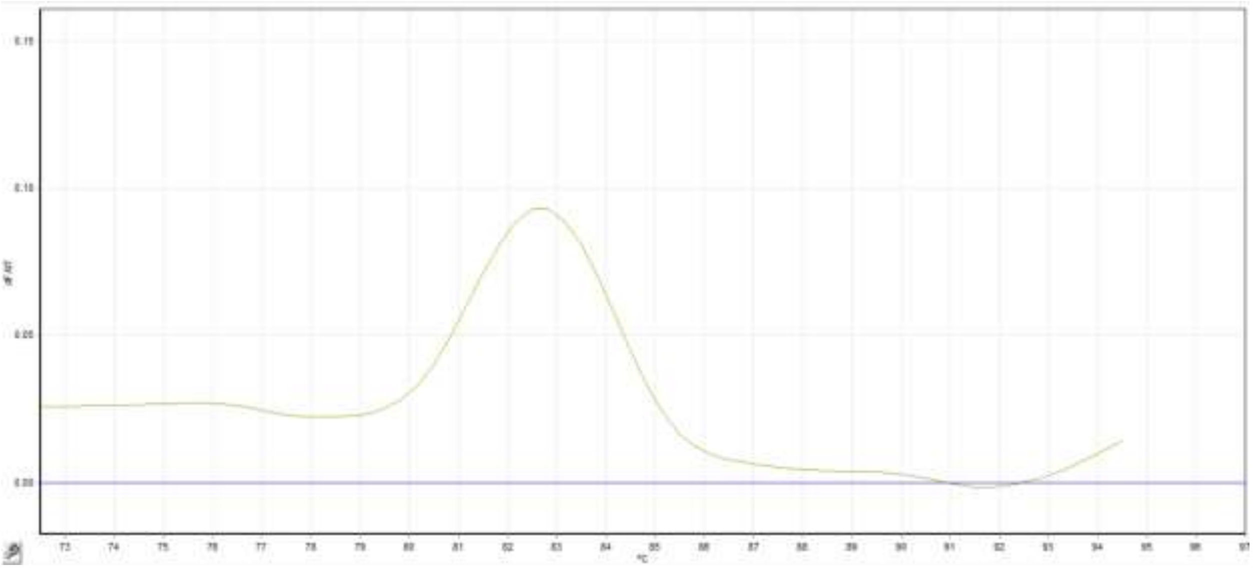

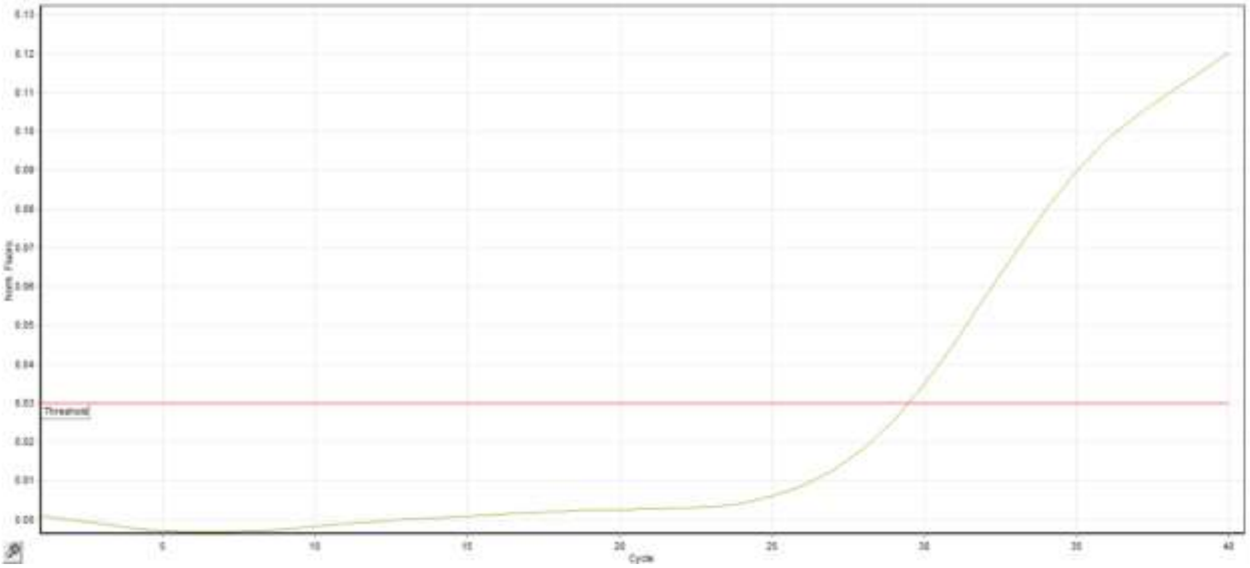

PGK1

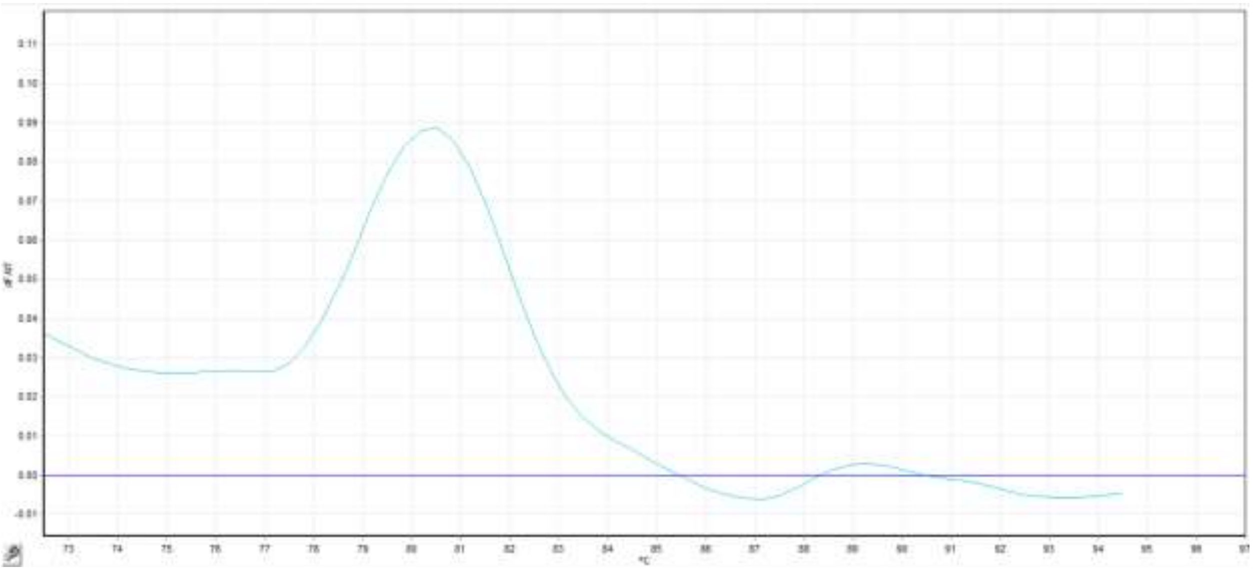

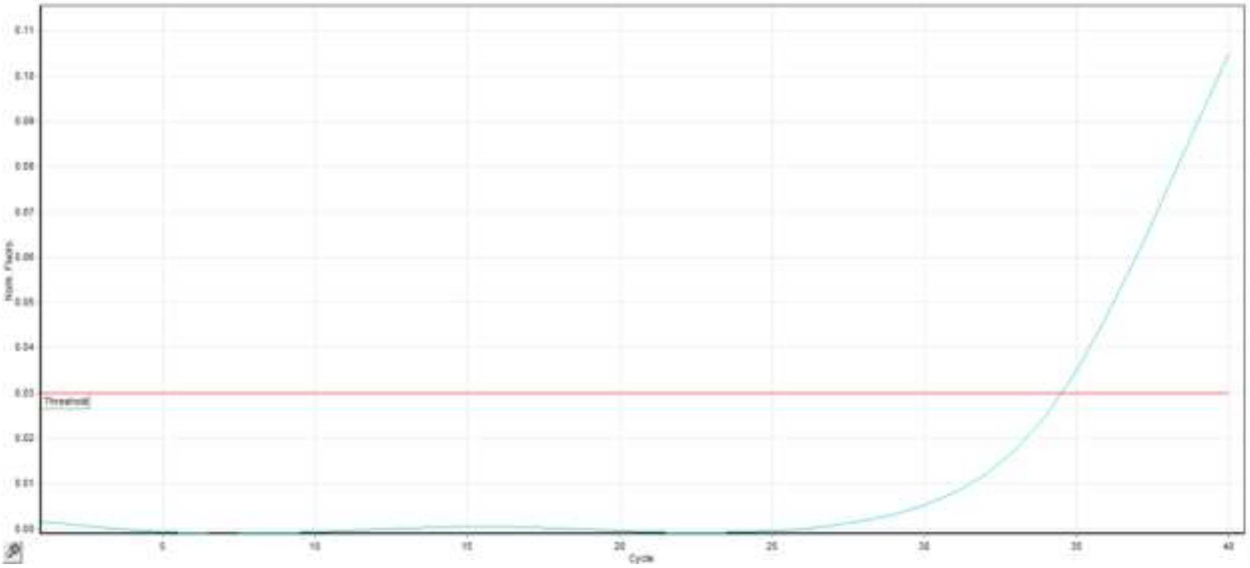

RPLP0

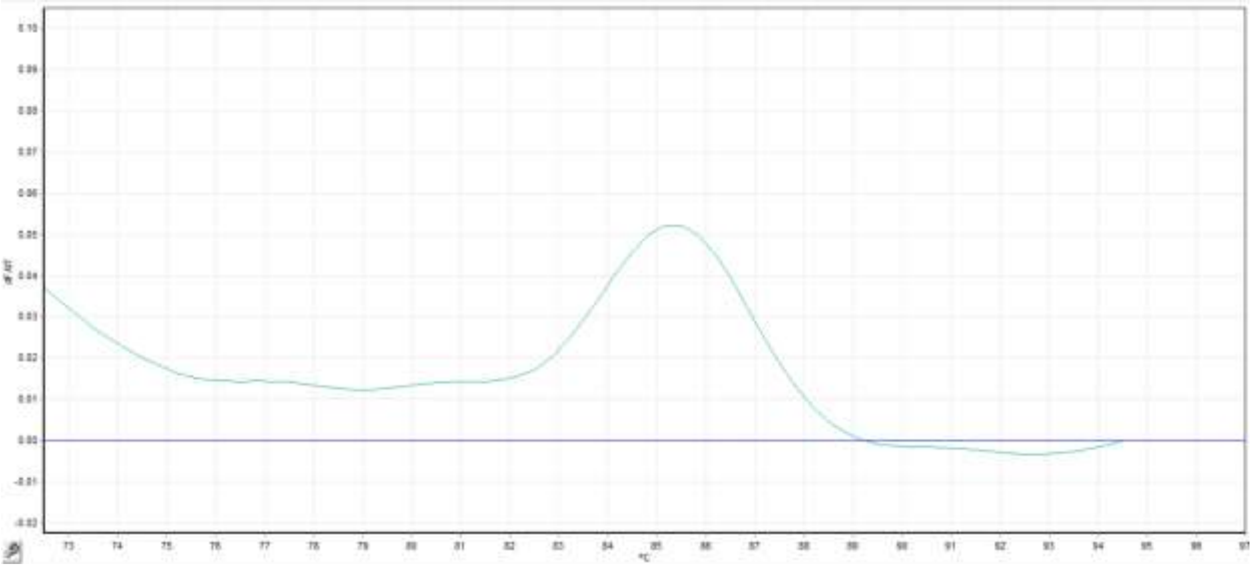

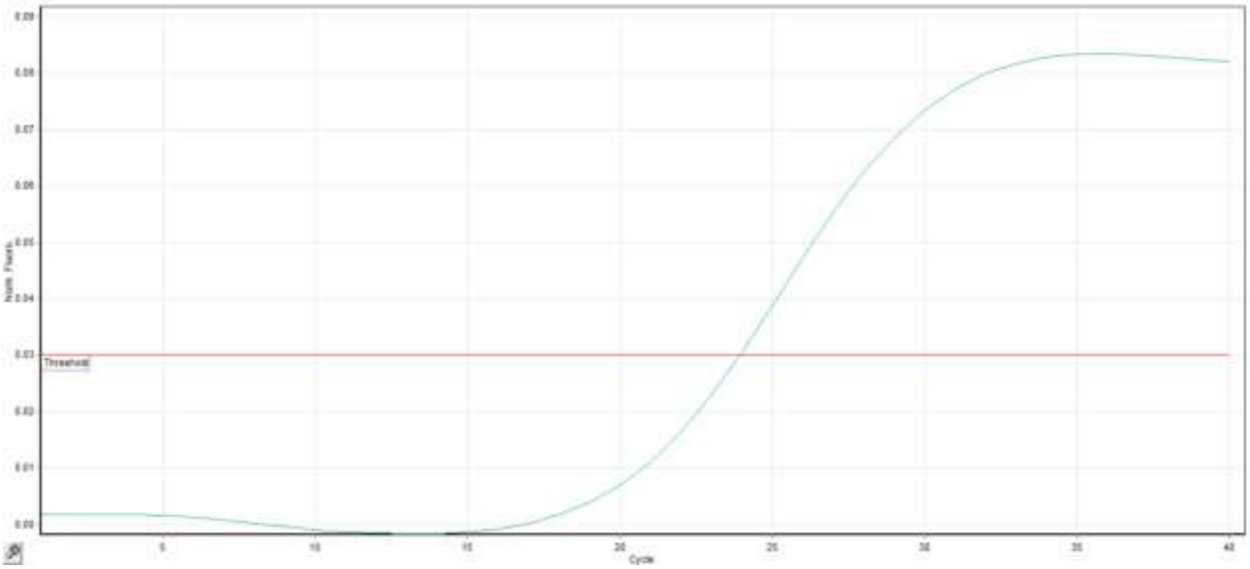

PGM1

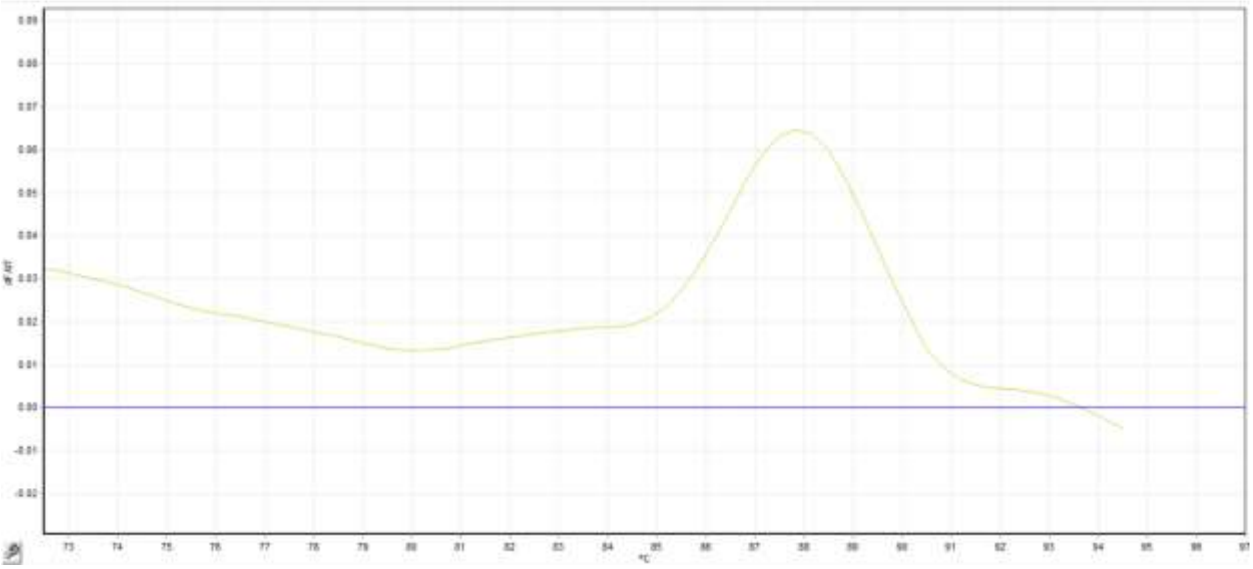

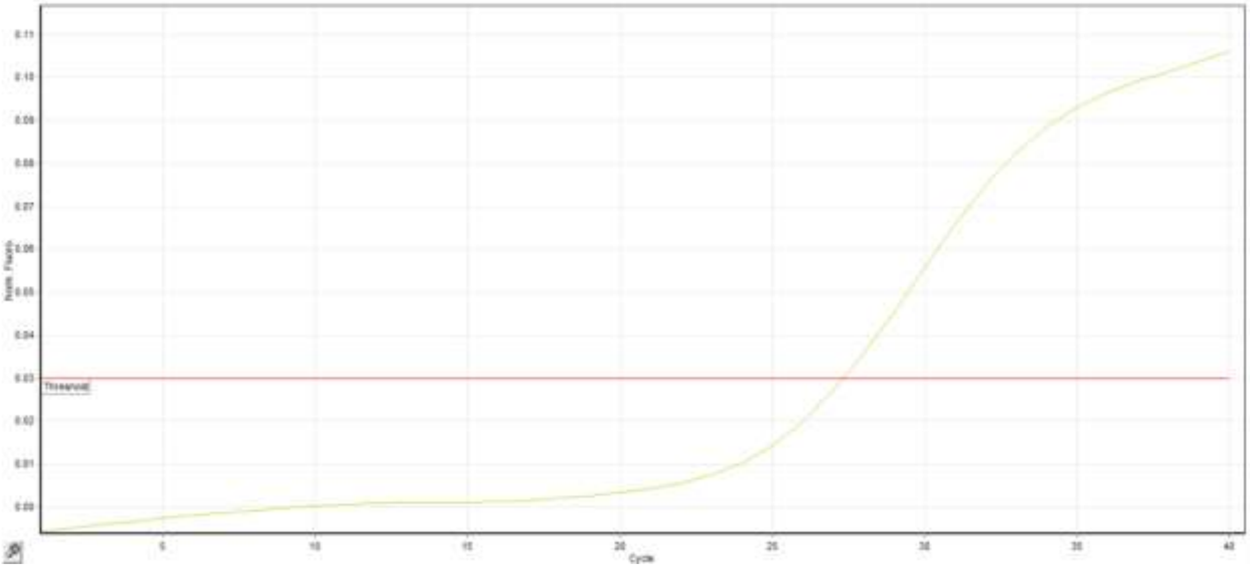

Supplement: Supplementary file 1 — Supplemental Figures [file 41598_2019_49247_MOESM1_ESM.pdf]
